# Formation of multiple G-quadruplexes contributes toward BCR fragility associated with chronic myelogenous leukemia

**DOI:** 10.1093/nar/gkaf167

**Published:** 2025-03-20

**Authors:** Shivangi Sharma, Elizabeth Thomas, Sumedha Dahal, Sayak Das, Shefali Kothari, Urbi Roy, Nitu Kumari, Vidya Gopalakrishnan, Sathees C Raghavan

**Affiliations:** Department of Biochemistry, Indian Institute of Science, Bangalore 560012, India; Department of Biochemistry, Indian Institute of Science, Bangalore 560012, India; Department of Biochemistry, Indian Institute of Science, Bangalore 560012, India; Department of Biochemistry, Indian Institute of Science, Bangalore 560012, India; Department of Biochemistry, Indian Institute of Science, Bangalore 560012, India; Department of Biochemistry, Indian Institute of Science, Bangalore 560012, India; Department of Biochemistry, Indian Institute of Science, Bangalore 560012, India; Department of Biochemistry, Indian Institute of Science, Bangalore 560012, India; Department of Zoology, St. Joseph's College, Irinjalakuda, Kerala 680121, India; Department of Biochemistry, Indian Institute of Science, Bangalore 560012, India

## Abstract

The Philadelphia chromosome, the translocation between *BCR* and *ABL* genes, is seen in 95% of chronic myeloid leukemia (CML) patients. Although discovered >60 years ago, the molecular mechanism of *BCR* fragility is unclear. Here, we have identified several G4 DNA motifs at the *BCR* fragile region of CML patients. Various lines of experimentation revealed that the breakpoint regions could fold into multiple intramolecular G-quadruplex structures. The sodium bisulfite modification assay revealed single strandedness in the fragile region when present on a plasmid and human genome. Circular dichroism spectroscopy revealed the parallel G4 DNA formation, leading to polymerase arrest at the *BCR* breakpoints. Intracellular recombination assay revealed that DNA breakage at the *BCR* fragile region could join with the break generated by ISceI endonuclease. Finally, purified AID could bind and deaminate cytosines when present on single-stranded DNA generated due to G4 DNA, both *in vitro* and inside the cells. Therefore, our results suggest that AID binds to G4 DNA present at the *BCR* fragile region, resulting in the deamination of cytosines to uracil and induction of DNA breaks in one of the DNA strands, which can later get converted into a double-strand break, leading to t(9;22) chromosomal translocation.

## Graphical Abstract

**Graphical Abstract:**
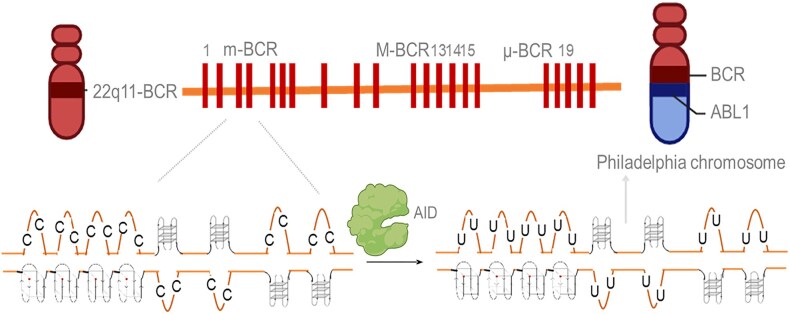

## Introduction

One of the significant threats to the genome and, human health is caused by chromosomal rearrangements [1–4]. Several non-B DNA structures, including cruciform, slipped structures, triplexes, G-quadruplexes, and Z-DNA, have been shown to cause genomic rearrangements such as mutations, deletions, expansions, and translocations in both prokaryotes and eukaryotes [3, 4]. Their distribution in the genome is not random and often co-localizes with sites of chromosomal breakage associated with genetic diseases [5, 6]. Various cancers are known to have chromosomal translocations as one of the most common genomic rearrangements [7–11]. The exchange of arms between the nonhomologous chromosomes leads to the chromosomal translocation, leading to oncogenesis [6, 12–16].

The Philadelphia chromosome was the first recurrent genetic alteration identified in association with a specific human cancer, chronic myeloid leukemia (CML) [17–21]. The Philadelphia chromosome is a shortened chromosome 22 resulting from a reciprocal translocation of parts of chromosomes 22 and 9. This translocation leads to a juxtaposition of protooncogene *c-ABL* (tyrosine kinase encoding gene on chromosome 9) and *BCR* on chromosome 22 [19]. Chromosome 22 is broken at band q11: chromosome 9 at band q34. The distal part of the long arm of chromosome 22 is attached to the broken end of chromosome 9, and the distal part of the long arm of chromosome 9 is attached to the broken end of chromosome 22. This configuration is designated as t(9;22)(q34;q11) translocation by standard nomenclature [18, 19].

The BCR-ABL fusion gene is commonly present in most patients with CML. It is also found in some patients with acute lymphoblastic leukemia (ALL) and acute myelogenous leukemia. All CML patients harbor the t(9;22) translocation [22, 23] and thus, it is a hallmark of CML. CML itself represents ∼15% of adult leukemias and has an incidence rate of 1–2 cases per 100 000 individuals [4, 24, 25].

The presence of non-B DNA structures in the genome is one of the major causes of genomic instability [2, 7, 13, 26–32]. G-quadruplex (G4) DNA is a non-B DNA structure that forms at guanine-rich genome regions. These structures could disrupt normal DNA processes like replication and transcription, contributing to genomic instability. Bioinformatic and BG4 ChIP analyses have revealed the presence of several such G4 DNA structures throughout the genome and in mitochondria [33–41]. G-quadruplex structures are four-stranded DNA molecules and are known to be present in the promoters of many genes and at the telomeres, thereby performing functions like gene regulation and chromosomal end protection [36, 38, 42–44]. Recent studies have also shown that altered DNA structures like cruciform, triplex DNA, and G-quadruplexes could play a significant role in the generation of chromosomal translocations in certain leukemias and lymphomas [3, 5, 13, 26, 28–30, 45–47].

Activation-induced cytidine deaminase (AID) is well known to promote somatic hypermutation and class switch recombination of immunoglobulin (Ig) genes in germinal center (GC) B cells [48]. It deaminates *Ig* gene cytosines into uracils, thereby creating DNA mismatches leading to mutations or DNA breaks [49–51]. Although AID primarily acts on Ig variables and switch regions, a few non Ig genes, like *BCL6*, c-*MYC*, *BCL11B*, *CD79A*, *CD79B*, *CD83*, and *PAX5*, are also shown to be targeted by AID in GC B cells [30, 47, 52–55]. This off-target activity of AID has been implicated in the malignant transformation of GC-derived B cell lymphomas and plasmacytomas [56–59]. Additional studies suggest that AID, together with RAG1/RAG2 enzymes, may also contribute to chromosomal translocations at early stages of B cell development [7, 28, 30, 47, 60]. The aberrant activity of AID and its ability to introduce deletions and chromosomal translocations combine to accelerate the progression from chronic phase into fatal B lymphoid blast crisis and drug resistance in patients with CML [61].

**Figure 1.**
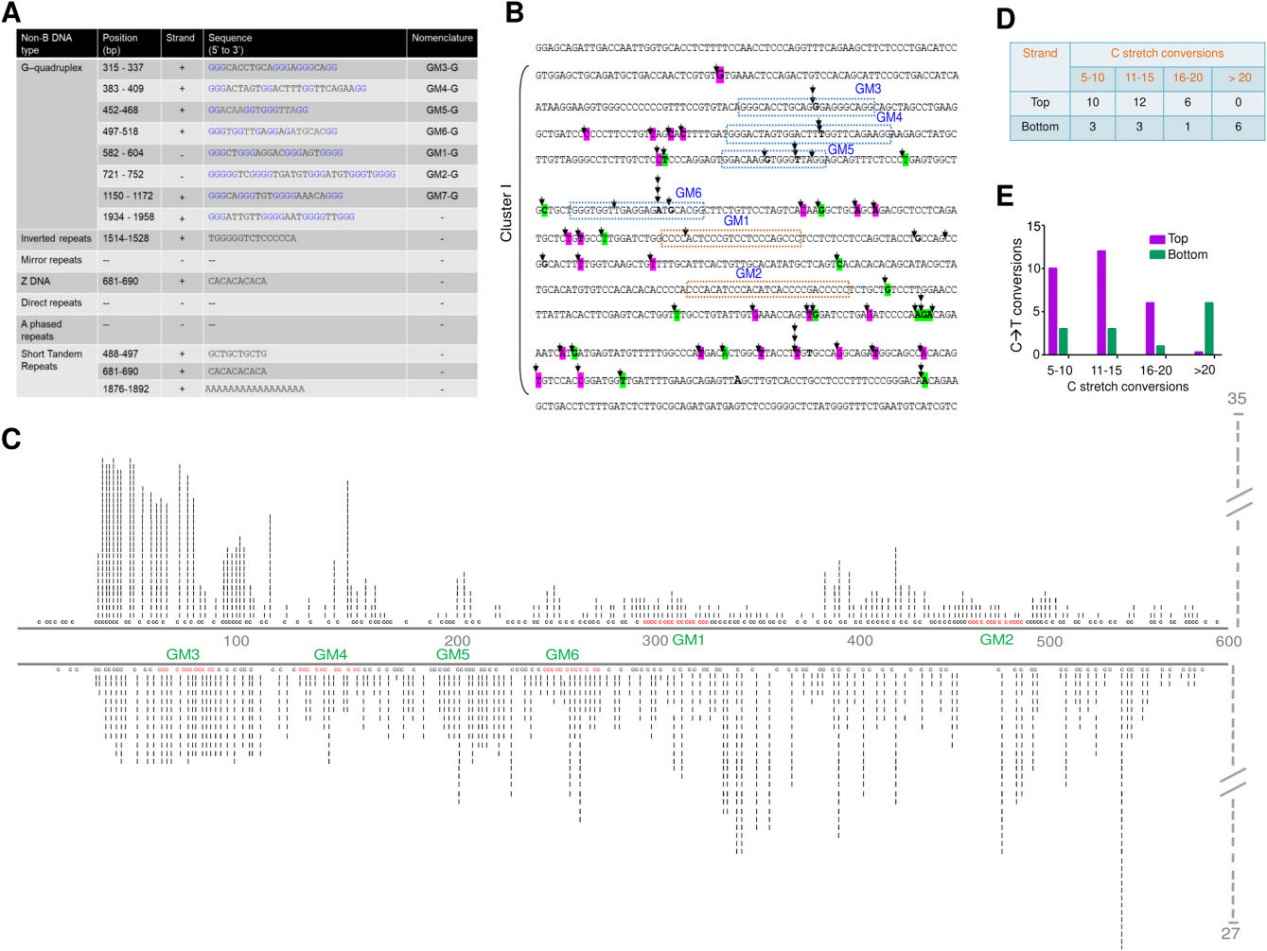
Analysis of *BCR* region for single-strandedness at the genome level. (**A**) Analysis of BCR breakpoint region for formation of different non-B DNA structures using NBDB and QGRS mapper database. (**B**) Schematic representation of the reported patient breakpoints in *BCR* Cluster I from CML patients (patient breakpoints were retrieved from GenBank: U19400.1). Dotted boxed region represents the predicted G-quadruplex region. The boxed region corresponding to GM3-GM6 indicates the formation in the (+) strand, while the boxed region corresponding to GM1-GM2 indicates the possible formation in the (−) strand. The black arrow represents the patient breakpoint regions. (**C**) Sodium bisulfite modification assay on chromosomal DNA extracted from K562 cells. The genomic DNA was isolated using non-denaturing conditions and subjected to sodium bisulfite modification assay. Each dark vertical line represents the number of times the particular cytosine in the *BCR* fragile region is converted to thymine after deamination in the presence of sodium bisulfite, followed by PCR and cloning. A total of 62 DNA molecules were sequenced from six different biological repeats. (**D**) Table represents the number of continuous C-stretch conversions. The calculation is based on the stretch conversion when 25 Cs have been analyzed. (**E**) Histogram representing the continuous conversion of cytosine to thymine in both the top and bottom strands..

In the present study, we investigated the possibility of the occurrence of non-B DNA structures at the BCR. We provide biochemical and biophysical evidence for multiple independent G-quadruplex structures at several reported patient breakpoints of BCR cluster I (Fig. 1A). We also provide proof of single-strandedness pertaining to multiple G-quadruplex motifs at the BCR Cluster I within the genome. These structures could block replication in a KCl-dependent manner on both single- and double-stranded DNA. More interestingly, mutations at the guanine stretch abolished the formation of the altered DNA structures on a double-stranded DNA *in vitro*. Finally, we show that AID can bind to *BCR* fragile regions, leading to the generation of DNA breaks. Thus, our study delineates mechanisms of fragility at the *BCR* during t(9;22) translocation.

## Materials and methods

### Enzymes, chemicals, and reagents

Chemical reagents were obtained from Merck Sigma (United States), and SRL (India). DNA-modifying enzymes were from New England BioLabs (United States) and Fermentas (United States). Radioisotope-labeled nucleotides were from BRIT (India). Culture media were from Sera Laboratory International Ltd (United Kingdom), and fetal bovine serum (FBS) and PenStrep were from Gibco BRL (United States).

### Cell lines and culture

The human myelogenous leukemia cell line K562 and the human pre-B cell lines Reh and Nalm6 were cultured in either RPMI 1640 with the medium supplemented with 10% FBS, 100 μg/mL penicillin, and 100 μg/mL streptomycin. The cells were maintained at 37°C in a humidified incubator with 5% CO_2_ [62, 63].

### Oligomeric DNA

The oligonucleotides used in the current study are listed in Supplementary Table S1. These oligomers were gel purified whenever required using 10–15% denaturing polyacrylamide gels as described [40], resuspended in TE, and stored at −20°C.

### 5′ end labeling of oligomers

5′- end labeling of oligomeric DNA was performed using T4 polynucleotide kinase in a buffer containing 20 mM Tris-acetate (pH 7.9), 10 mM magnesium acetate, 50 mM potassium acetate, 1 mM dithiothreitol, and [γ- P^32^] ATP at 37°C for 1 h [64]. The labeled substrates were purified using a G-25 Sephadex size exclusion column and stored at -20°C until further use.

### Mapping of breakpoint junctions

The sequence of BCR gene on chromosome 22 was retrieved from NCBI and about 120 patient breakpoint junctions were mapped using databases viz., TIC db, db CRID, NCBI and previous literature [65].

### *In silico* analysis for G-quadruplex formation

The BCR gene was analysed for presence of non-B DNA structure motifs using non-B DB database, G4 Hunter [40, 66] and QGRS mapper as described earlier [33, 37, 67, 68]. The parameter for the number of repeated guanines was kept at two or three to assess the possibility of forming both 2G and 3G plate quadruplex structures.

### Gel mobility shift assay

The radiolabeled oligomers were incubated in the presence of 100 mM KCl in Tris-EDTA (TE) buffer (pH 8.0) at 37°C for 1 h [40, 69, 70]. Different forms of G-quadruplexes were then resolved on 12–15% native polyacrylamide gels in the presence of 100 mM KCl, both in the gel and the electrophoresis buffer, at 150 V at room temperature (RT). The gels were dried and exposed to a screen, and signals were detected using phosphorImager FLA9000 (Fuji, Japan).

### Electrophoretic mobility shift assay (EMSA) for determining DNA-protein interactions

To determine whether AID binds to G-quadruplex-forming regions, radiolabeled oligomers derived from BCR clusters I and II (GM2, GM3), a mutant oligomer, and a random oligomer (Random) were heat denatured in 100 mM KCl and slowly cooled to allow G-quadruplex formation [47, 71, 72]. Increasing concentrations of GST-tagged AID protein (20, 40 and 60 nM, respectively) were incubated with 20 nM DNA. After incubating at 4°C for 1 h, the complexes were resolved on 6% native polyacrylamide gels prepared (19:1 acrylamide-to-bisacrylamide ratio), with 100 mM KCl in the gel and running buffer, dried, and visualized using a phosphorImager, FLA9000.

### Circular dichroism spectroscopy

The *BCR* GM2 region and GM3 wild-type, mutant, and C-rich complementary oligomers were incubated either in the presence or absence of 100 mM KCl in TE at 37°C for 1 h as described previously [26, 29, 40, 47, 73]. The circular dichroism (CD) spectra were recorded at RT from 200 to 300 nm and 10 cycles were accumulated for every sample, using a JASCO J-810 spectropolarimeter at a scan speed of 50 nm/min. A separate spectrum was measured for buffer alone for five cycles, and this was subtracted from all experimental spectra. For destabilization of the G-quadruplex structure, spectra were recorded for samples incubated with KCl at 90°C. Further, a CD spectrum was recorded after the structure was reformed by incubating the same sample overnight at 37°C. The ellipticity was calculated using the Spectra Manager software and plotted as a function of wavelength.

### Taq polymerase stop assay

Taq polymerase stop assay was performed as described previously (Nambiar at al., 2011). The BCR oligomers for GM2, ET19 [wild type], ET20 [double mutant], oligomers for GM3 region, ET21 [wild type] and ET22 [double mutant]), random oligomer (MN117) [26], and the HIF1α promoter region (MN118) [26] were heat denatured and slowly reannealed in the presence of 100 mM KCl with radiolabeled MN96 or MN119 (for HIF1 α). After the addition of 200 mM dNTPs and 1 U Taq polymerase, the reactions were incubated at 47°C for 30 min. The products were resolved on a 12% denaturing polyacrylamide gelelectrophoresis (PAGE) and visualized as described above.

### Dimethyl sulfate protection assay

Dimethyl sulfate (DMS) protection assay was performed as described previously [26, 40, 70]. The radiolabeled oligomers were incubated in TE in the presence of 100 mM KCl at 37°C for 1 h. DMS was added to the reaction mixture (1/200 dilution) and incubated for 15 min at RT. An equal volume of piperidine (10%) was added to each tube, and the reaction mixture was incubated at 90°C for 30 min. The reaction was diluted to double the volume and the vacuum dried. The pellet was further washed with water thrice and dried using a speedvac concentrator. The reaction products were resolved on an 18% denaturing polyacrylamide gel, dried and visualized.

### Site-directed mutagenesis

The construction of mutants at *BCR* GM2 and GM3 regions was performed by site-directed mutagenesis using specific primers containing the desired mutation as described previously [41, 74]. Two sets of primers (ET15-ET31 and ET32-ET16) were used to amplify *BCR* GM2 containing mutations in the first round of polymerase chain reaction (PCR). The PCR products from these two reactions were then mixed and served as the template for the second round to get the region II amplification product using the primers ET15 and ET16. Similarly, two sets of primers (ET17-ET27 and ET28-ET18) were used to amplify the GM3 containing the mutations, followed by a second round of PCR using ET17-ET18 to get region III amplification product. These PCR products were cloned and checked by restriction enzyme digestion, followed by sequencing.

### Plasmid construction

pET3 was constructed by PCR amplification of the BCR cluster 1 (GM1 and GM2) from the human genomic DNA and cloned into the SalI site of pMN2, in the physiological orientation. The mutant plasmid, pET8, was generated using site-directed mutagenesis. It contains a 3 nt mutation wherein two stretches of guanines are converted to adenines and thiamine. The pET8 contains the mutant sequence of the BCR region that was cloned into the SalI site of pIRES2 EGFP. pET2 was constructed by cloning the BCR wild-type region III into the SalI site of pMN2, while pET7 contains the mutated GM3 at the SalI site of pMN2.

### Primer extension

The presence of replication blocks due to G-quadruplex structure formation [41, 75] at the BCR breakpoint, regions GM1, GM2 and GM3 were studied in plasmids pET3 and pET8 by primer extension. The reactions were carried out by mixing 100 ng of DNA sample in 1X Thermo polymerase buffer [10 mM KCl, 10 mM (NH4)_2_SO_4_, 20 mM Tris-HCl (pH 8.8), 4 mM MgSO_4_ and 0.1% Triton X-100], 200 μM deoxynucleoside triphosphates (dNTPs), 0.5 μM end-labeled oligomers, and 1 U Vent (exo-)polymerase. Linear amplification primer extensions were carried out in a PCR machine (20 cycles) under the following conditions: 95°C for 3 min (1 cycle), 94°C for 45 s, 58 to 64°C for 45 s (as specified), 72°C for 45 s, and final extension for 3 min. The annealing temperatures of the primers used were 58°C for ET33 and ET34 for ET36 and ET38. The reactions were terminated by adding a formamide dye, and products were resolved on an 8% denaturing polyacrylamide gel. The gel was dried, and signals were detected using phosphorImager. Sequencing reactions using respective primers were carried out for the wild-type plasmids and loaded along with the primer extension reactions on the gel.

### *In vivo* recombination assay

The recombination assay was performed as described earlier [74, 76, 77]. Nalm6 or Reh cells were transfected with appropriate episomal substrates, by electroporation and cultured for 48 h at 37°C. The plasmid substrates were recovered by using the rapid alkaline lysis method and used for transforming *Escherichia coli* DH10β. The transformation mixture was plated on ampicillin (A) and chloramphenicol-ampicillin (CA) LB agar plates. The recombination frequencies (R) were calculated using the equation (CA/A) x 100). Each eukaryotic transfection was typically analyzed with multiple *E. coli* transformations.

### Sodium bisulfite modification assay

The sodium bisulfite assay was performed as described previously [30, 73, 74, 78]. Briefly, chromosomal DNA was isolated from K562 (chronic myelogenous leukemia) cell line using the nondenaturing method. Both pET3 plasmid and genomic DNA were subjected to bisulfite modification treatment. Approximately 5 μg of DNA was incubated in 12.5 μl of 20 mM hydroquinone and 457.5 μl of 2.5 M sodium bisulfite (pH 5.2) for 14–16 h at 37°C and purified using the Wizard DNA cleanup kit (Promega, Madison, WI). The bisulfite-modified DNA was desulfonated with 0.3 M NaOH at 37°C for 15 min, ethanol precipitated and resuspended in 25–30 μl of TE buffer. The BCR breakpoint region (GM2 and GM3) from the bisulfite-treated DNA was PCR amplified, resolved on an agarose gel, purified, TA cloned, and sequenced.

### AID overexpression and purification

For the overexpression and purification of AID protein, BL21 (DE3) cells were transformed with pGEX_5X-3GST:hAID expression vector, induced by the addition of 1 mM IPTG and incubation for 16 h at 16°C. Cells were harvested and lysed in a buffer containing 1X PBS, 500 mM NaCl, 1% Triton-X, and PMSF (1 mM) [30]. After lysis, the supernatant was loaded on glutathione-sepharose beads, and GST AID protein was eluted using 10 mM of glutathione. The purity and identity of the protein were confirmed using sodium dodecyl sulfate (SDS)-PAGE, followed by western blotting.

### Western blotting

To verify the identity of the purified protein, approximately 20 μg of lysate, was resolved using 10% SDS-PAGE to perform the western blotting [47, 76]. Following gel electrophoresis, the proteins were transferred onto a PVDF membrane (Merck Millipore). The membrane was blocked with 5% skimmed milk powder for 1 h at RT. The blocked membrane was then incubated overnight at 4°C with an anti-GST antibody (1:500 dilution, B-14, SC-138, Santa Cruz). The following day, the membrane was incubated with a biotinylated secondary antibody (1:10 000 dilution, Santa Cruz) for 2 h at RT. After extensive washing with PBST, the membrane was further incubated with streptavidin-HRP (1:10 000 dilution, Sigma) for 30 minutes at RT. Finally, the blots were developed using a chemiluminescent detection reagent (Immobilon™ Western, Millipore) and visualized using a chemiluminescence imaging system (LAS 3000, Fuji).

### Chromatin immunoprecipitation assay

Chromatin immunoprecipitation was performed with slight modifications [47, 79, 80]. Briefly, 4 × 10^6^ K562 cells were cultured and grown for 24 h at 5% CO_2_, 37°C incubator. Cells were then crosslinked with formaldehyde in a final concentration of 1% and quenched by adding 100 μl of 1.375 M glycine per milliliter of culture. Cells were then pelleted down, washed with 1X PBS, and subjected to cell lysis buffer (5 mM PIPES, 85 mM KCl, 0.5% NP40) and then nuclei lysis buffer (50 mM Tris pH 8, 10 mM EDTA, 1% SDS). Lysed samples were then sonicated (Diagenode biorupter) with 30 s on/ 45 s off pulse for 30 cycles. Sonicated chromatin was stored at −80°C for a minimum of 8 h. For chromatin preclearing and immunoprecipitation, the chromatin was thawed in ice and centrifuged at high speed for 15 min. The supernatant was collected, and DNA purity (A_260_/_280_) was measured using nanodrop. Samples were divided into beads control, input, secondary control, and experimental. Relevant antibodies were added to the sample and allowed to bind and incubated for 8–10 h. Protein A/G-agarose beads (sigma) were added to the chromatin samples and incubated for 2 h. The beads were pelleted down and washed with high salt buffer (50 mM HEPES (pH 7.9), 500 mM NaCl, 1 mM EDTA, 0.1% SDS, 1% Triton X-100, 0.1% deoxycholate) five times and further washed twice with TE. The beads were then incubated in elution buffer (50 mM Tris [pH 8.0], 10 mM EDTA, 1% SDS) and Proteinase K (20 μg/μl) at 55°C for 2 h, and cross-linking was reversed by incubating overnight at 65°C. Finally, DNA was purified by phenol:chloroform and precipitation. The DNA obtained was amplified using PCR for BCR regions that support the formation of G-quadruplex structure and different control regions that do not support any secondary structure formation.

### Statistical analysis

For statistical analysis, a two-tailed Student's t-test was performed using GraphPad Prism (Version 5.1; San Diego, CA, USA) to determine the significance of the experimental results. A p-value of less than 0.05 was considered statistically significant. Data are expressed as mean ± SEM.

## Results

### Analysis of the *BCR* fragile region reveals the formation of multiple G-quadruplex structures

When the *BCR* gene was analyzed for patient breakpoints, it was observed that most of the breakpoints were in a 1.7 Kb *BCR* fragile region [81, 82] (Fig. 1B and Supplementary Fig. S1). To ease the analysis, the region was divided into three clusters (Clusters I, II, and III) based on the clustering of patient breakpoints. When the region was evaluated using a non-BDB database, G4Hunter [40, 66, 67], and QGRS mapper [68], we identified eight distinct short regions containing G-quadruplex sequence motifs (Fig. 1A). Among these, four regions had stretches of three guanines (identified by non-BDB database, QGRS, and G4Hunter), and other four regions contained stretches of two guanines (identified by QGRS and G4Hunter). Additionally, we detected one inverted repeat and three short tandem repeats (Fig. 1A). Since most predicted motifs are present in Cluster I, we have analyzed this region extensively in this study.

Formation of non-B DNA could result in single-strandedness on the complementary strand or at the junctions where structural variation occurs. To elucidate the single-strandedness of Cluster I, a bisulfite modification assay was performed on chromosomal DNA from K562 cells (Fig. 1C). Results revealed that Cluster I has a high tendency of cytosines to uracil conversion, revealing single-strandedness, which suggests a possibility of the existence of secondary structures like G-quadruplexes, cruciform, etc. Analysis of 62 clones from Cluster I (595 bp; Fig. 1C) revealed that 35 clones were from the top and 27 from the bottom strand. Among the clones from the top strand, 6 had stretch conversions of 16–20 Cytosines, 12 clones had 11–15 C-stretch conversions, and 10 clones had 5–10 C-stretches conversions. Similarly, among the clones from the bottom strand, 6 clones had conversions in more than 20 cytosine stretches, 1 had conversion in 16–20 C-stretches, 3 had 11–15, and 3 had conversions in 5–10 C-stretches (Fig. 1C-E). These results suggest that significant levels of single-strandedness exist in BCR Cluster I.

### The G-rich sequence of the BCR cluster I can fold into multiple G-quadruplexes

**Figure 2.**
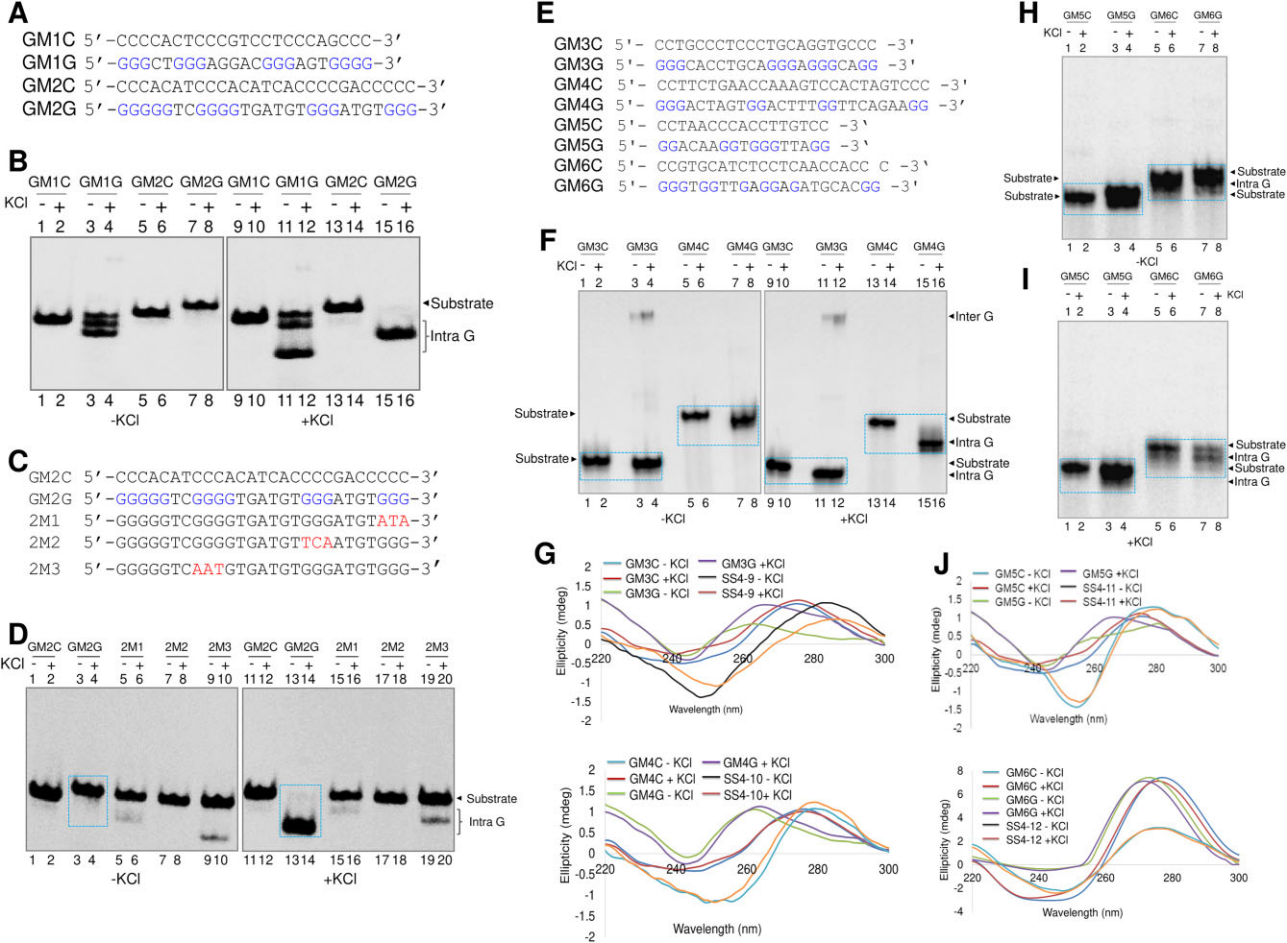
Investigation of G-quadruplex formation from predicted *BCR* region. (**A**) The oligomeric sequence of the G-rich strand (indicated by “GM1G” and “GM2G”) and complementary C strand (indicated by “GM1C” and “GM2C”) of both GM1 and GM2 derived from the *BCR* Cluster I. (**B**) The G- and C-strands from both regions were incubated in the presence of KCl and resolved in the absence and presence of KCl (100 mM) both in the gel and electrophoresis buffer. The substrate, intramolecular (Intra G) quadruplex structures are indicated. (**C**) The oligomeric sequence of the C-rich (indicated by “GM2C”), G-rich strand for region II (indicated by “GM2G”) and its mutants (indicated by “2M1,” “2M2,” “2M3”) derived from the *BCR* Cluster I. (**D**) The C-rich, G-rich and mutants were incubated in the presence of KCl (100 mM) and resolved in the absence and presence of KCl in the gel and electrophoresis buffer. The intramolecular (Intra G) quadruplex structures are boxed. (**E**) The oligomeric sequences of the G-rich strand (indicated by “GM3G, GM4G, GM5G, and GM6G”) and complementary C-strand (indicated by “GM3C, GM4C, GM5C, and GM6C”) derived from the *BCR* Cluster I predicted to form 2G plate quadruplex structures from the nontemplate strand. (**F**) The G and C strands (GM3 and GM4) were incubated in the presence of KCl (100 mM) and resolved in the absence and presence of KCl in the gel and electrophoresis buffer. (**G**) CD spectra for both the regions (GM3 and GM4) are shown for both C and G strands and control oligonucleotides (SS4-9 and SS4-10, which have similar GC content as GM3 and GM4 but lack stretches of guanines) in TE without and with incubation in KCl at 37°C for 1 h. (**H** and **I**) The G and C strands (GM5 and GM6) were incubated with and without KCl (100 mM) and resolved in the absence (**H**) and presence (**I**) of KCl in the gel and electrophoresis buffer. (**J**) CD spectra for the regions GM5 and GM6 are shown for C and G strands. CD spectra for control oligonucleotides (SS4-11 and SS4-12), which have similar GC content as GM5 and GM6 but lack stretches of guanines, are shown. Oligomeric DNA was resuspended in TE and incubated in KCl at 37°C for 1 h. “In the presence of KCl” indicates that KCl is present in the gel and running buffer.

To check whether the G-rich sequences predicted using a non-BDB database have the potential to fold into the non-B DNA structures *in vitro*, we designed the oligomeric DNA spanning the short region representing the “G” strand and the complementary “C” strand (Fig. 2A, C, and E). γ-P^32^ATP -labeled oligomeric DNA were incubated in the presence of KCl and resolved on native polyacrylamide gels with and without KCl in the gel and buffer. Results showed that both C-rich and G-rich substrates migrated according to the molecular weight in the absence of KCl in the gel and buffer (Fig. 2B, D, F, H, and I). Importantly, when the same substrates were electrophoresed in the presence of KCl in the gel and buffer, we observed G-rich strands from GM1 and GM2 migrating much faster than the C-rich strands (Fig. 2B). The faster-moving species represented the formation of intramolecular G-quadruplex. However, there was no difference in the mobility of DNA species on native gel, when samples were prepared irrespective of the presence of KCl (Fig. 2B, D, F, H, and I).

Interestingly, we observed the presence of three bands when GM1G oligomeric DNA was resolved on a native PAGE, of that one of the bands moved faster when KCl was added to the gel and running buffer (Fig. 2B). This suggests that the DNA might be in multiple conformational states. The band that remained unchanged even after adding KCl could be the unfolded DNA, while the other two bands may represent different conformations of the intramolecular G4 DNA that are stabilized differently when KCl was added. Interestingly, there was no intermolecular G-quadruplex formation in either of the cases and all the species could fold into intramolecular G-quadruplex. The structure was abrogated when the oligomers containing mutated G-stretches were used for the study (Fig. 2C and D).

In addition, we performed a gel shift assay to check whether the predicted G-rich sequences spanning 2G repeats could fold into a G-quadruplex structure (Fig. 2E, F, H, and I). We observed that the region corresponding to GM4G showed the formation of a strong intramolecular G-quadruplex. In addition, the region corresponding to GM3G could fold into intermolecular G-quadruplex even in the absence of KCl in the gel and buffer (Fig. 2F). However, the region corresponding to GM5G and GM6G showed the presence of mixed species (both intramolecular species and substrate) suggesting weaker G-quadruplex formation (Fig. 2H and I).

Further, circular dichroism spectroscopy was performed to study the structural conformation of DNA. Interestingly, GM2G showed a characteristic peak at 290 nm and a dip at 260 nm (Supplementary Fig. S2A), indicating the formation of antiparallel G-quadruplex irrespective of KCl. To determine whether the structure gets destroyed at higher temperatures, CD spectra of GM2G were recorded at increasing temperatures in the presence of KCl (Supplementary Fig. S2B). CD spectra of GM2G region began to collapse at temperatures above 60°C leading to complete destruction of G4 structure at 90°C (Supplementary Fig. S2B). However, the restoration of the G-quadruplex structure was observed after the renaturation of GM2G sequence at 37°C (Supplementary Fig. S2C). Similarly, when the other G-quadruplex forming oligomers predicted to form two-plate G-quadruplex was subjected to CD, irrespective of the presence of KCl, all the four regions showed a peak at 260–265 nm and dip at 240–245 nm, In contrast, the corresponding C-strand oligonucleotides and another oligomeric DNA with similar GC content, but lacking stretches of guanines were used as controls, which showed a typical peak for ssDNA at 275–280 nm and dip at 240–245 nm (Fig. 2G and J).

### G4 DNA formation at the BCR fragile region prevents the DNA polymerase progression when present on a plasmid DNA

**Figure 3.**
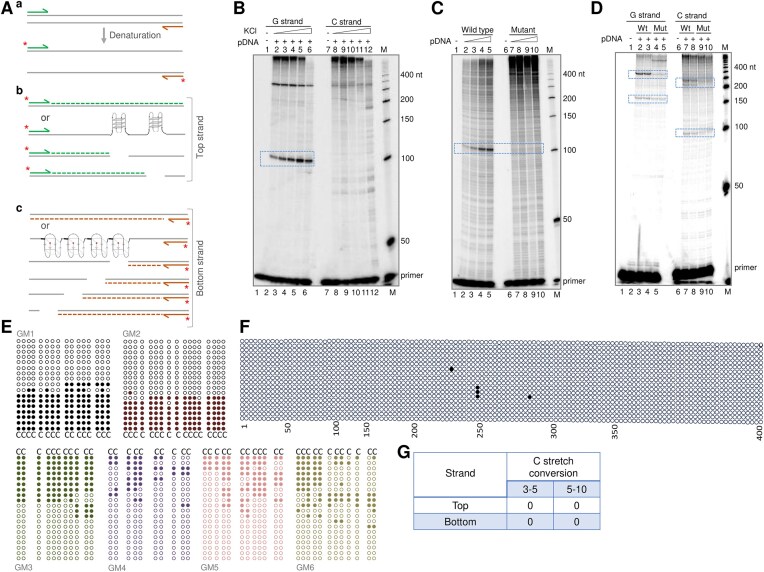
Evaluation of replication pause sites and the single-strandedness due to G4 DNA formation at GM2 region of the *BCR* breakpoint when present within a plasmid DNA. (**A**) Schematic representation of primer extension assay on plasmid DNA (a) or top (b) and bottom strand (c). Two G-quadruplex structures are predicted in top strand (b), while four structures in the bottom strand (c). Either of these structures may result in pause sites upon primer extension. (**B**) The primer extension reactions for plasmid pET3 harboring *BCR* breakpoint region were performed in the presence of increasing concentrations of KCl (0, 10, 20, 50, 100 mM) using the primers ET33 for the G strand (lane 1–6) and ET34 for the C strand (lanes 7–12). The pause site is marked with a boxed region. (**C**) A comparison of replication pause induced on pET3 (wild-type plasmid containing the *BCR* breakpoint region) (lanes 1–5) and pET8 (mutant of *BCR* breakpoint region) (lanes 6–10) following primer extension reactions using radiolabeled primers for the G-rich strand (ET33). An increasing concentration of plasmid DNA (12.5, 25, 50, and 100 ng) was used for the primer extension study. The extension reactions were done for 20 cycles in a buffer containing KCl. The replication pause site and anticipated pause sites are boxed. (**D**) Primer extension on GM2 region of pET3 (lanes 2, 3, 7, and 8) and pET8 (lanes 4, 5, 9, and 10) using primers (ET36 and ET38) that bind at different positions with respect to the G-quadruplex-forming motif. In all panels, “M” denotes the radiolabeled 50 nt ladder. (**E**) Sodium bisulfite modification assay on pET3 plasmid containing the *BCR* breakpoint region. The plasmid DNA was subjected to sodium bisulfite modification assay. Each dark circle represents the respective cytosine in the *BCR* breakpoint regions converted to thymine after deamination in the presence of sodium bisulfite, followed by PCR. A total of 25 DNA molecules were sequenced, out of which around five molecules were entirely unreactive for sodium bisulfite treatment. The cytosines in GM1-GM6 sequences of *BCR* are indicated using different colors. (**F**) Representative bisulfite sensitivity for the top strand of 400 bp region control region from pET3 plasmid. Each row of circles represents a single DNA molecule (a clone). A total of 18 clones were sequenced for control. Each dark circle represents the respective cytosine in the control regions converted to thymine after deamination in the presence of sodium bisulfite. (**G**) The table shows the number of C-stretch conversions of the control region presented in panel F.

In order to check whether the G-quadruplex structure can form in the context of double-stranded supercoiled DNA, we cloned the *BCR* region (700 bp) in a plasmid, pET3. The formation of G-quadruplex DNA could block the DNA polymerase progression during replication. Primer extension was performed as described before [75] using radiolabeled primers positioned at varying distances upstream and downstream of BCR G-rich region II (GM2) at the top and bottom strand (Fig. 3A). Interestingly, we observed a prominent pause site at 110 nt position corresponding to the G-quadruplex forming sequence in the G-rich strand in a KCl dependent manner (Fig. 3B). The intensity of pause site in the G-rich strand increased with increase in KCl concentration (Fig. 3B, lanes 2–6). Importantly, when the primer extension assay was performed on the same plasmid, pET3, using the primer for the complementary C strand, such a pause site corresponding to the G4 motif was absent (Fig. 3B, lanes 7–12). This result further reinforces the formation of G-quadruplex DNA at the BCR region when present on a plasmid DNA.

Since the formation of G-quadruplex structures flanking the *BCR* region could block the progression of polymerase, we wondered whether mutations to the stretches of guanines at region II could facilitate the polymerase progression. A 3 nt (G-A/T) mutation at two stretches of G’s was introduced using a site-directed mutagenesis approach. The mutant plasmid, pET8, was constructed by cloning the mutant sequence, replacing the *BCR* GM2 region. Primer extension was performed using the mutant plasmid, pET8, to test the effect of mutation on the progression of polymerases. Interestingly, we observed that the mutated G-rich sequence could not block the polymerization, and hence, there was a complete absence of the pause site, which resulted in the generation of the full-length primer extension product (Fig. 3C, lanes 6–10). Thus, the mutation at the two G stretches of the GM2 region in the *BCR* blocked the G4 DNA formation, emphasizing the role of G-quadruplexes in imparting fragility to this region.

Further, we performed primer extension on wild-type plasmid, pET3, and the mutant plasmid pET8 using radiolabeled primers, ET36 and ET38 positioned at different locations from GM2 of *BCR* (Supplementary Fig. S3A). Results showed the presence of a prominent pause site at or adjacent to G4 motifs. The pause sites were observed around 150 and 300 nt, corresponding to GM5 and GM6, respectively (Fig. 3D, lanes 2 and 3), whereas in the mutant plasmid, pET8, there was reduced pause site observed owing to the lack of G4 structure formation (Fig. 3D, lanes 4 and 5).

Besides, when the complementary C strand was analyzed in both the wild-type and mutant plasmids, pause sites were detected at approximately 100 nt and 250 nt positions. In the wild-type plasmid, these pauses were more pronounced (Fig. 3D, lanes 7 and 8), attributing to the complementary sequences of G4 motifs of GM1 and GM2. Notably, the intensity of these pause sites was significantly less in the mutant plasmid (Fig. 3D, lanes 9 and 10). Considering that the C-rich sequences could fold into i-motif structures, the observed pause sites are not surprising [83, 84]; however, they warrant further investigation. Thus, our results suggest that forming a G-quadruplex structure on a plasmid DNA blocked the progression of DNA polymerases through the BCR fragile region.

Further, we performed a Taq polymerase stop assay to test whether the G4 DNA forming sequence of the BCR region could induce a pause during the progression of DNA polymerase. In the wild-type G-rich strand of the GM2 region (WT1), we observed a major pause site corresponding to the G-quadruplex forming sequence, irrespective of the presence of the K^+^ ion (Supplementary Fig. S3B and C lanes 2 and 3). Upon mutating the two stretches of Gs of WT1 (Mut1), the pause site intensity was significantly reduced when KCl was absent in the reaction, and we could observe a full-length extension product (Supplementary Fig. S3C, lane 4). Contrary to this, a strong pause site was observed in the presence of KCl (Supplementary Fig. S3C, lane 5). It is possible that upon mutating two stretches of guanine, the remaining G stretches in GM2 region could fold into intermolecular structures and were stabilized by K^+^ ions. Further, the wild-type G-rich strand of GM3 (WT2) exhibited two pause sites at the corresponding G-quadruplex forming sequence in the presence of KCl (Supplementary Fig. S3C, lane 7), whereas, upon mutation of the guanines from GM3 (Mut2), there was a complete disappearance of pause sites and simultaneous appearance of full-length extension products independent of K^+^ ion (Supplementary Fig. S3C, lanes 8 and 9). When a random sequence (RN) with a similar GC content was tested, it showed no pause site, reinforcing the role of guanine stretches in the structure formation (Supplementary Fig. S3C, lanes 10 and 11). The G-quadruplex forming region from the HIF1α promoter was used as a positive control (Supplementary Fig. S3C, lanes 13 and 14). Thus, our results confirm the G-quadruplex-induced polymerase block at the *BCR* fragile region

### Single-strandedness exists at the *BCR* breakpoint cluster region in plasmid DNA

Since we observed structure formation in the G-rich strand of the plasmid DNA, we were interested in testing the single-strandedness of the complementary strand. To do this, a sodium bisulfite modification assay was performed after isolating plasmid DNA comprising the breakpoint cluster region using nondenaturing method [7, 26]. Following sodium bisulfite treatment, the fragile region was PCR amplified, cloned, and sequenced. When a cytosine is present in the single-stranded DNA, it can react with bisulfite, leading to deamination and conversion to uracil, which, upon PCR amplification, can be read as cytosine to thymine conversion, providing information on single-strandedness in the parental sequence. Results revealed that out of the 25 clones sequenced, 20 molecules exhibited significant C to T conversion at different extents (Fig. 3E). The C to T conversions observed in most of the sequenced clones indicate that the complementary C-rich strand does not consistently form a stable double-stranded helix, probably due to the G-quadruplex formation on the opposite G-rich strand within the BCR region. Further, we have also analyzed a control region from the backbone of the same plasmid using SS4-5 and SS4-6 primers. The sequencing of 18 clones from the control region revealed no significant C→T conversions (Fig. 3F and G), thereby confirming that bisulfite reactivity is specific to regions due to G4 DNA formation. This suggests that single-strandedness exists at the fragile BCR region in CML, driven by the G-quadruplex structure in the G-rich sequence.

### BCR fragile region undergo recombination within the cells

**Figure 4.**
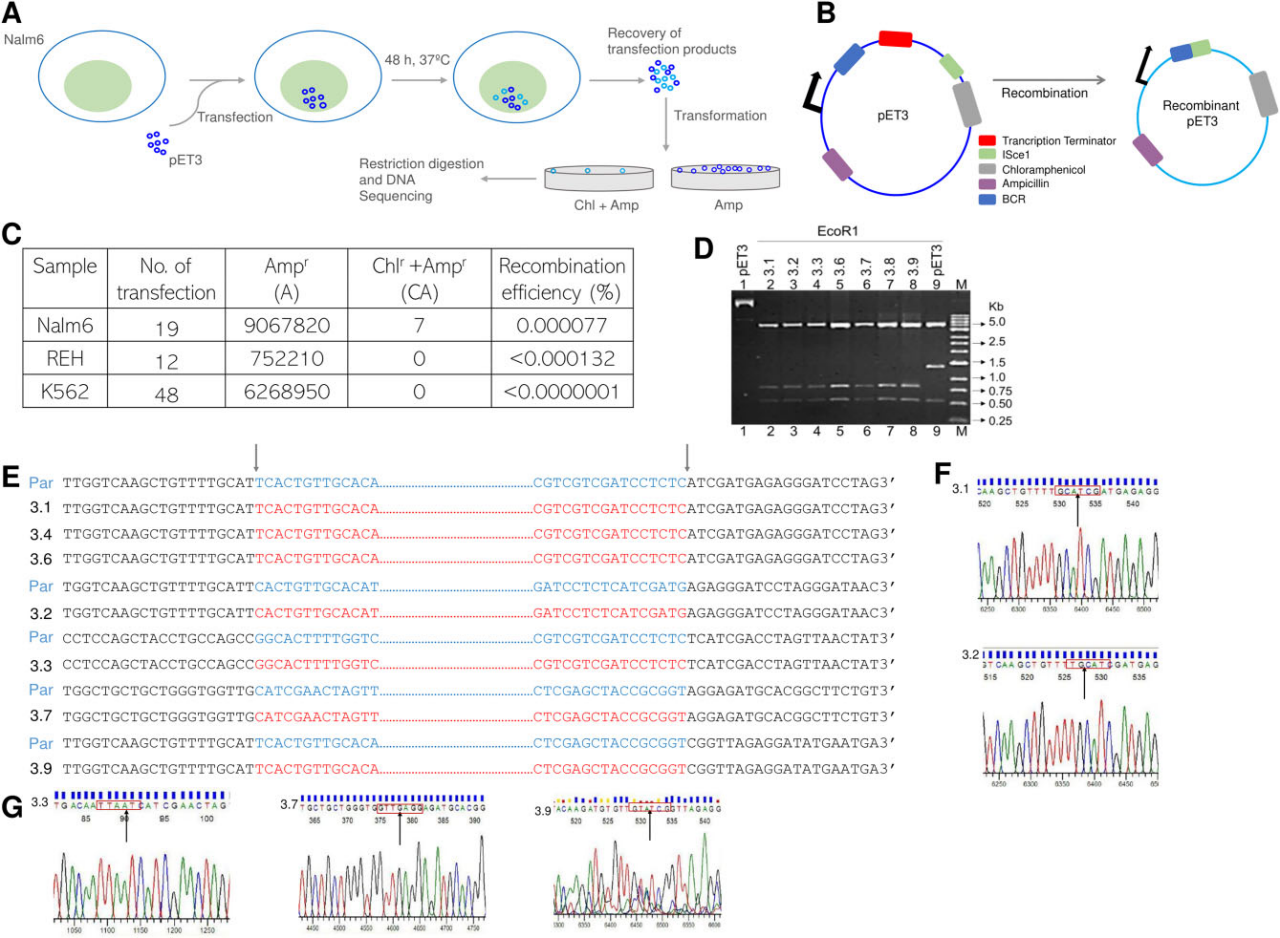
Intracellular translocation assay for detecting *BCR* recombinants. (**A**) Schematic representation of the episomal substrates (pET3) used for intracellular translocation assay. pET3 was transfected by electroporation into Nalm6, Reh, and K562 cells, incubated for 48 h, and recombinants were recovered. The episomal DNA purified from cells was transformed into *E. coli* DH10β. Following electroporation, the transformation mix was plated on Amp (**A**), and Chloramphenicol-Ampicillin (CA) plates and the recombinants were confirmed by restriction digestion followed by DNA sequencing. (**B**) Schematic representation of the episomal substrates used (pET3) and the expected recombinant upon transfection. (**C**) Table summarizing the analysis of clones and recombination frequency in Nalm6, Reh, and K562 cells. The number of colonies obtained on ampicillin (**A**) and chloramphenicol-ampicillin (CA) selective media for different transfection batches are shown in the table. The recombination frequency (Rf) is calculated by the formula: (CA/A)*100. (**D**) Agarose gel profile showing products obtained after EcoRI digestion of the recombinant clones. M denotes 1 kb ladder. (**E**) Following DNA sequencing of the recombinant clones, a total of seven recombinant episomes were obtained where *BCR* genes underwent recombination. Of the seven, three showed recombination junction exactly at the same nt, while others were independent clones with breakpoint regions at different places. The positions of the missing nucleotide sequences are indicated distinctly, while the parental (par) sequences are represented using distinct notations. The vertical arrow indicates sequence at the break site in each clone. (**F** and **G**) A segment of the chromatogram showing the breakpoint region in the case of each clone. In the figure, the recombined sequence is boxed.

Since we observed the formation of G-quadruplex on oligomeric DNA, and plasmid DNA, we were interested in testing whether the formation of G4 DNA inside the cells could contribute to the fragility of the *BCR* region. Intracellular translocation assay was performed by transfecting episomal construct, pET3. For this, pET3 was constructed by cloning the *BCR* fragile region and ISceI site such that they are separated by a transcription termination site [85]. The episomal construct was then electroporated along with the ISceI expression vector into lymphoid cell lines, Nalm6, Reh, and K562 cell lines (Fig. 4A and B). Following the transformation of the transfection products, the double resistant (chloramphenicol and ampicillin) recombined products were evaluated (Fig. 4C). Results showed the occurrence of multiple recombinants when the episome was transfected inside the Nalm6 cells; no recombinants were obtained in Reh and K562 cell lines. These results suggest a significant difference in the recombination potential among these cell lines. The confirmation of the recombinants in Nalm6 was carried out by restriction digestion of these recombinants using EcoRI (Fig. 4D) enzyme followed by DNA sequencing (Fig. 4E–G). The recombination frequency was estimated to 0.000077% in Nalm6, while it was <0.000132% in Reh and <0.0000001% in K562 (Fig. 4C). The absence of recombinants in Reh and K562 could be attributed to several factors. One possibility could be the inherent differences in the repair mechanisms or chromatin structure among these cell lines. In case of K562, which is derived from a CML patient, may have a distinct chromosomal architecture due to the presence of the BCR-ABL fusion gene. Additionally, the ability of these cells to process and resolve G-quadruplex structures may differ, affecting the likelihood of recombination events. Physicochemical factors, such as intracellular ionic conditions (e.g. potassium ion concentration), may play a critical role in stabilizing G-quadruplex structures, while macromolecular factors, including the expression and activity of helicases (e.g. BLM, WRN) and nucleases, are essential for processing these structures during replication and repair [86]. Furthermore, variations in DNA repair pathway utilization, such as homologous recombination versus nonhomologous end joining, can also influence the ability of cells to resolve G-quadruplex-induced DNA breaks. However, the recombination in Nalm6 suggests that G-quadruplex structure formation inside the cells leads to the DNA breakage and recombines with the double-strand break induced by ISceI endonuclease. DNA break occurred at different positions downstream of the G-quadruplex forming region I (GM1) and II (GM2) (Fig. 4). A total of seven recombinants were obtained (Fig. 4E). Overall, these results provide evidence for fragility at the G-quadruplex forming motifs of the BCR inside the cells, which further supports its role in chromosomal translocation events leading to chronic myelogenous leukemia.

### AID can bind to *BCR* fragile region both within the cells and *in vitro*

**Figure 5.**
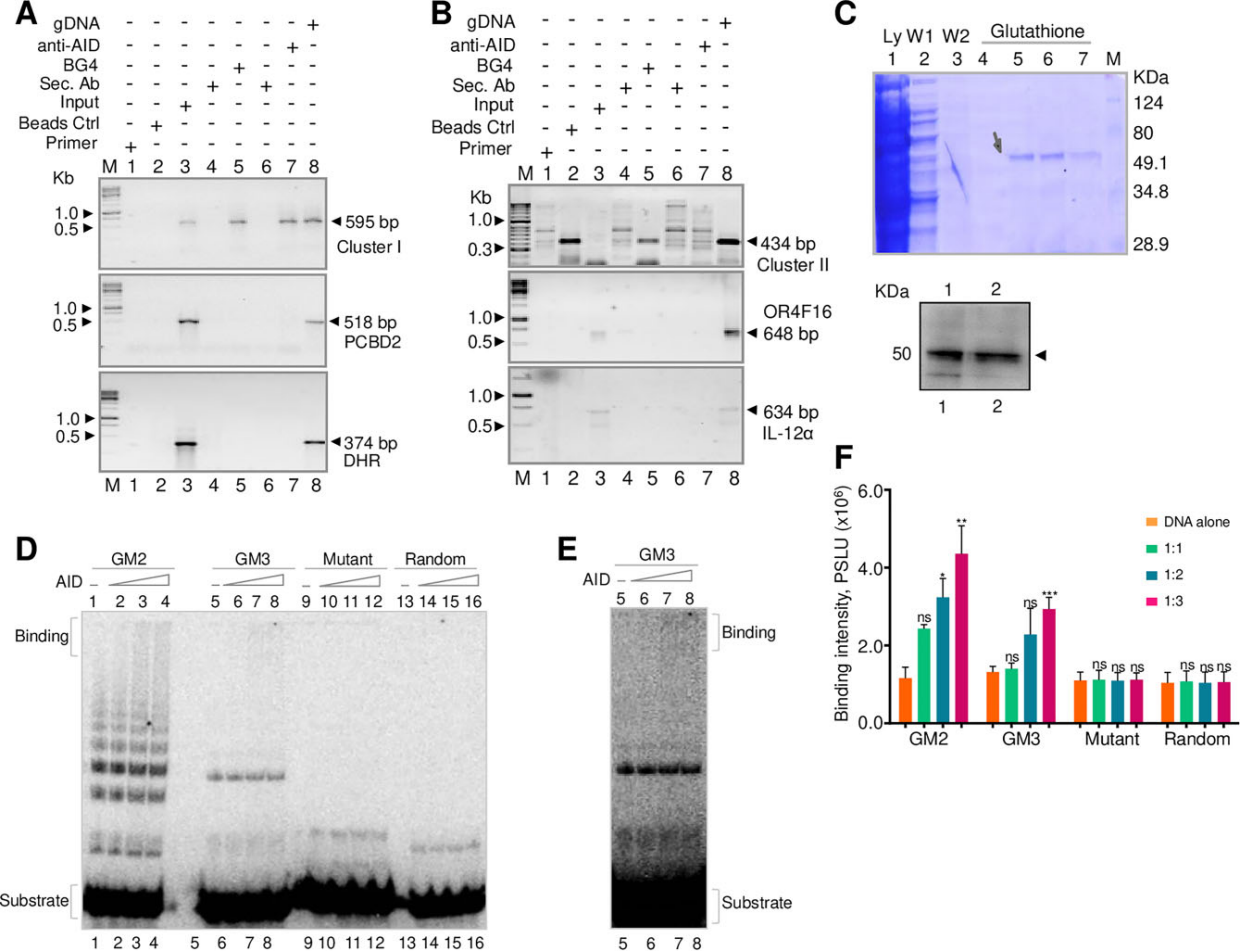
Evaluation of AID binding to *BCR* fragile region within cells and *in vitro*. (**A**) ChIP analysis using anti-BG4 and anti-AID to evaluate binding on AID to *BCR* Cluster I and BG4 antibody to G4 DNA. PCR amplification of BG4 ChIPed DNA and AID ChIPed DNA for *BCR* Cluster I containing G-quadruplex forming motifs and different control regions (*PCBD2* and *DHR*) which do not contain any G-quadruplex forming motifs. (**B**) PCR amplification of BG4 and AID ChIPed DNA for *BCR* Cluster II containing G-quadruplex forming motifs and different control regions (OR4F16 and IL-12α) which do not contain any G-quadruplex forming motifs. In both the panels, lane 1 corresponds to no template control, lane 2 is beads control, lane 3 is Input DNA, lane 4 is rabbit IgG antibody control, lane 5 is BG4 pulldown, lane 6 is goat IgG antibody control, and lane 7 is AID pulldown in all the gels. ‘M’ represents the DNA marker, and the expected amplification product has been specified. The input DNA served as the positive control, Sec. Ab, and beads control as negative controls in all the cases. (**C**) Overexpression and purification of AID protein by transforming BL21 (DE3) cells with pGEX_5X-3GST: hAID expression vector. Cells were harvested and lysed. The supernatant was loaded on glutathione-Sepharose beads, and GST-AID protein was eluted using 10 mM of glutathione and loaded (lanes 5–7) on SDS-PAGE. Ly denoted the lysate, W1 and W2 are the wash 1 and 2; the arrow represents the band of purified AID protein. The identity of the protein was confirmed by Western blotting using an anti-GST antibody. The arrow indicates the AID band, which is tagged with GST. (**D**) EMSA studies were done by incubating purified AID with BCR G-quadruplex-forming regions (GM2, GM3), a mutant oligomer, and a random control region lacking G4 DNA motif (Random). Increasing concentrations of AID (20, 40, and 60 nM) were added to the substrate DNA (20 nM) (DNA to protein ratios of 1:1, 1:2, and 1:3, respectively) and incubated at 4°C for 1 h. (**E**) A higher exposure of the gel containing GM3 is presented. A higher exposure of the whole gel is presented in Supplementary Fig. S4C. (**F**) Following gel electrophoresis, the AID binding was quantitated (indicated as PSLU units), and the graph was created using GraphPad Prism 5.0. The error bar was calculated as mean ± SEM (“ns” indicates not significant, *P < 0.05: **: *P* ≤ 0.01, ***: *P* ≤ 0.001).

AID is a single-stranded (ss) DNA deaminase that converts cytosine to uracil and generates diversity within the immunoglobulin loci during class switching and somatic hypermutation [87–89]. When paired with deoxy guanosine, this converted deoxy uracil residue is mutagenic as it mimics thymidine during DNA replication. Apart from this, AID is also known to bind the G-quadruplex structures and create lesions in the sequence [90]. To determine whether AID binds *BCR* fragile region within the cells, we performed a chromatin immunoprecipitation assay. Additionally, we have also used BG4 antibody [35, 36, 91] to detect G4 DNA formation within the cells in the same assay. Specific enrichment of Clusters I and II was seen in the samples subjected to ChIP (ChIPed) using BG4 and AID antibodies, suggesting the binding of AID to Clusters I and II of BCR regions. (Fig. 5A and B). Importantly, the binding of BG4 to the BCR fragile region further confirms the formation of G4 DNA structure (Fig. 5A and B). In contrast, no amplifications were observed when four different random regions were used. The random regions were chosen in such a way that they cannot fold into secondary structures or are not known to bind to AID. The absence of amplification in the random regions thus confirms the specificity of BG4 binding to the G-quadruplex structures *in vivo* [35, 36, 91]. More importantly, the observed binding of AID reveals its possible role in imparting fragility to the BCR breakpoint region. Secondary antibody and beads-only samples were also amplified to check for the specificity of the proteins. Input DNA and genomic DNA were used as positive controls. The AID and BG4 ChIPed DNA from BCR cluster I was sequenced to confirm the identity of the fragment (Supplementary Fig. S4A and B).

Further biochemical assays were performed to evaluate the possible role of AID in making the *BCR* region fragile. To do this, GST-AID protein was overexpressed and purified from pGEX_5X-3GST: hAID expression vector (Fig. 5C). The purity and identity of the protein was confirmed using SDS-PAGE followed by western blotting (Fig. 5C) and the activity of AID protein was confirmed by AID-mediated deamination reaction [30, 54, 92, 93]. To determine whether AID can bind to the G-quadruplex forming regions, DNA oligomers derived from BCR cluster I and II (GM2, GM3), a mutant oligomer, and a random oligomer (Random) were incubated with increasing concentrations of purified AID protein (20, 40, and 60 nM). We observed an increase in the binding intensity with increasing concentration of protein. In contrast, such binding was absent when only buffer was added (Fig. 5D and Supplementary Fig. S4C, lanes 1–8, and 5D–F), suggesting that purified AID can bind to G-quadruplex structures, albeit with poor binding efficiency. When a mutant and random oligomer that cannot fold into a G-quadruplex structure was incubated with purified AID, there was no binding observed (Fig. 5D and E and Supplementary Fig. S4C, lanes 9–16; 5F). Thus, our results suggest that AID binds to the G-quadruplex DNA structure both *in vitro* and inside the cells.

### Two plates G4 DNA are formed at BCR Cluster II

**Figure 6.**
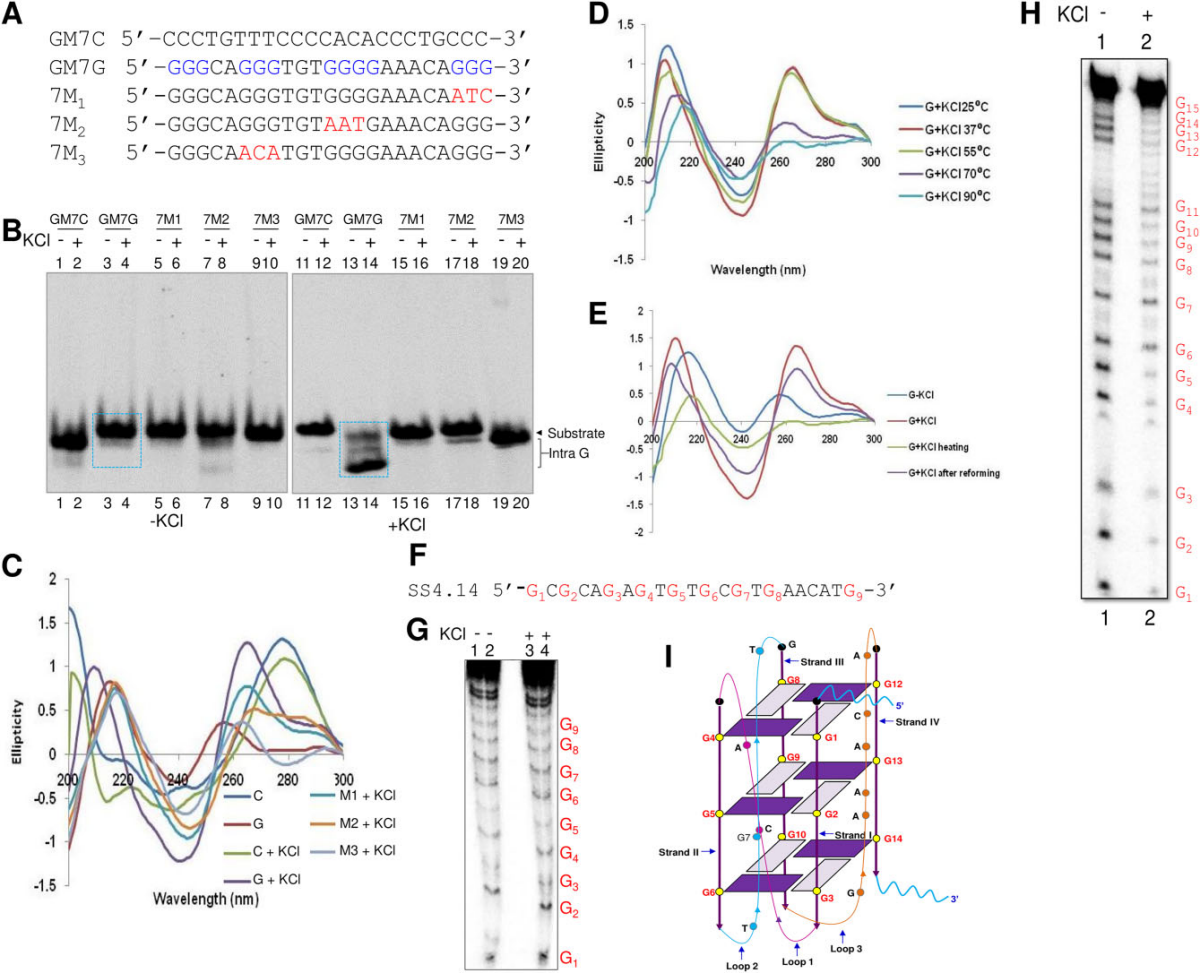
Evaluation of the G-quadruplex formation at the Cluster II of the *BCR* breakpoint region. (**A**) The oligomeric sequence of the C-rich strand (indicated by “GM7C”), G-rich strand (indicated by “GM7G”), and its mutants (indicated by “7M1,” “7M2,” “7M3”) of GM3 derived from the *BCR* region Cluster II. (**B**) The G-stretch and mutants were incubated in the presence of KCl (100 mM) and resolved in the absence and presence of KCl in the gel and electrophoresis buffer. The intramolecular (Intra G) quadruplex structures are boxed. “In the presence of KCl” indicates KCl is present in the gel and running buffer. (**C**) CD spectra for G strand of GM7 and its mutants in TE with and without KCl incubated at 37°C with a scan range of 200–300 nm. (**D**) CD spectra for the G-strand of the GM7 region in TE in the presence of KCl at 25°C, 37°C, 55°C, 70°C, and 90°C. (**E**) CD spectra for the G-strand of the GM7 region in TE with KCl at 37°C for 1 h along with heat-denatured oligomer and reformation of the structure. (**F**) The sequence of the control oligomeric DNA used for the DMS protection assay. (**G**) DMS protection assay for control oligomeric DNA. The radiolabeled DNA strand was incubated in the presence and absence of 100 mM KCl at 37°C for 1 h, followed by treatment with DMS and cleavage with piperidine, and were resolved on a 15% denaturing PAGE. (**H**) DMS protection assay for GM7 region of *BCR* breakpoint region. The wild-type G-rich strand was incubated in the presence and absence of 100 mM KCl at 37°C for 1 h, followed by treatment with DMS and cleavage with piperidine. (**I**) A representative two-dimensional model of the intramolecular G-quadruplex structure was formed at the *BCR* cluster II. The position of guanines and other nucleotides involved in the structure are marked. Arrows indicate the orientation of the strands.

Since the results so far revealed the formation of G4 DNA structure at Cluster I, we were interested in testing the status of G4 DNA in Cluster II, where we identified a single G4 DNA forming motif based on *in silico* studies (Fig. 1A). Besides, we had also noted that AID and BG4 can bind to Cluster II following the ChIP PCR (Fig. 5A and B). To do this, the oligomeric DNA spanning the region (GM7) representing the “G” strand and the complementary “C” strand was designed (Fig. 6A, Supplementary Fig. S5A). [P^32−^γ] ATP-labeled oligomeric DNA was incubated in the presence and absence of KCl and resolved on native polyacrylamide gels with and without KCl in the gel and running buffer. Results showed that both C and G substrates migrated according to the molecular weight in the absence of KCl in the gel and running buffer, while G-rich-strand of Cluster II (GM7) migrated much faster than the C-rich strands (Supplementary Fig. S5B). The faster-moving intramolecular G-quadruplex formation was abrogated when the oligomers containing mutated G-stretches were used (Fig. 6A and B). This observation was further supported by CD spectroscopy where GM7 exhibited a parallel G-quadruplex with a characteristic peak at 260 nm and a dip at 240 nm (Fig. 6C). The structure formation in the cluster II was abolished when CD studies were performed at higher temperatures (70°C and 90°C) in the presence of KCl (Fig. 6D). However, the G-quadruplex structure was restored when allowed to renature at 37°C (Fig. 6E).

In order to decipher the nature of the base pairing of guanines during the formation of G-quadruplex, we performed a DMS protection assay [26, 41, 73]. During G-quadruplex formation, the guanines are involved in Hoogsteen base pairing. Therefore, the N7 position is not free for methylation and hence does not react with DMS [26]. We used the wild-type G-rich sequence of GM7 regions and a control random sequence in the presence and absence of KCl for the DMS protection assay (Fig. 6F–H). In the case of GM7, we observed that except G17, all the other guanines were involved in the G-quadruplex formation, when KCl was present (Fig. 6H, lane 2), whereas all the guanines showed equal reactivity in the absence of KCl (Fig. 6H, lane 1). In contrast, the control oligomeric DNA (SS4-14), which lacks stretches of guanines, showed no protection from DMS in the presence or absence of KCl (Fig. 6G).

Further, DMS protection assay on oligomer derived from GM2 region showed DMS protection in all the guanines except G7, when KCl was present confirming the G-quadruplex formation (Supplementary Fig. S6A and B). However, the protection observed for G12 and G13 was marginally less pronounced than other guanines, suggesting that these positions may experience partial exposure or participate in a less stable Hoogsteen base pairing. This could be due to the structural dynamics of the G-quadruplex, where G12 and G13 may be at the edges or in regions of the structure prone to transient conformational flexibility. This observation aligns with the results of the gel mobility shift assay, which demonstrated that all species of wild-type G-strands of GM2 could fold into intramolecular G-quadruplex DNA (Fig. 2).

Based on the guanines involved in the G-quadruplex formation, we proposed a two-dimensional model (Fig. 6I, and Supplementary Fig. S6C). GM2 region could form an antiparallel G-quadruplex, comprising three G-tetrads, G3, G10, G14, G20; G4, G9, G15, G19 and G5, G8, G16, G18 (Supplementary Fig. S6C), whereas GM7 could form a parallel G-quadruplex comprising three G tetrads, G3, G6, G10, G14; G2, G5, G9, G13 and G1, G4, G8, G12 (Fig. 6I).

## Discussion

### The formation of G-quadruplexes in the BCR fragile region explains its fragility during the generation of the t(9;22) translocation

In the current study, we have identified several G-rich motifs that can independently adopt either parallel or anti-parallel intramolecular G-quadruplex structures in *BCR* fragile regions. These structures’ formation depended on KCl and was abrogated when even a single G-stretch was mutated. The structure formation could also block replication in both oligomers and on a plasmid. Evidence for the structure formation on genomic DNA was provided by sodium bisulfite modification assay, where several molecules showed the presence of full or partial single-strandedness. Thus, our data demonstrate the existence of multiple independent G-quadruplexes in the *BCR* fragile region.

A number of studies have linked the role of different non-B DNA structures like G-quadruplexes, cruciforms, and R-loops to the fragility of genomic regions involved in chromosomal translocations [13, 26–28, 30, 46, 94–97]. Previous studies have suggested that the RAG complex could recognize and generate a nick at a region proximal to the G-quadruplex structure in case of t(14;18) translocation [3, 7, 26, 29]. Similarly, in the case of t(11;22)(q23;q11), the presence of cruciform structures has been shown to form at palindromic AT-rich repeat (PATRR) sequences on chromosome 11 [98]. Besides, the breakpoint region in the c-*MYC* gene was shown to form G-quadruplexes and its involvement in the t(8;14) translocation in Burkitt's lymphoma [30, 99]. The present study observed that the breakpoint lies before the G-quadruplex-forming motifs at Cluster I through the *in vivo* recombination assay. Further studies are warranted to investigate the recombination efficiency of other breakpoint clusters.

### Regulation of G-quadruplexes inside cells

In order to facilitate the formation of non-canonical structures, a double-stranded DNA must unwind into the single-stranded form, which occurs during physiological processes like replication and transcription. The formation of such structures could be highly stable, however, several factors such as the presence of enzymes like resolvases, helicases, or higher temperature could affect the structure formation [100]. The obliteration of the structure was observed when CD was performed at higher temperatures (above 60°C) for GM2 region, while for GM3, the structure seemed to be highly stable as the higher temperature did not affect its stability. Our primer extension studies showed that these structures block polymerases progression, that could lead to replication stalling and, thus, the introduction of a nick, which could later get converted into a double-strand break. The presence of such breaks may result in translocations if the structure formation is not resolved. Similarly, previous studies have shown that the formation of such alternate structures makes the neighboring region fragile due to the presence of nucleases as in the case of RAGs at the G-quadruplex site in *BCL2 MBR* [7, 26], MRE11 and DNA2 helicase on either side of the G-quadruplex structure *in vitro* [101]. Therefore, an alternate possibility for the generation of a break at these structures could be due to recognition by structure-specific nucleases followed by their cleavage at G4 DNA, replication stalling, or nucleotide misincorporation during repair.

### Mechanism of t(9;22) translocation

**Figure 7.**
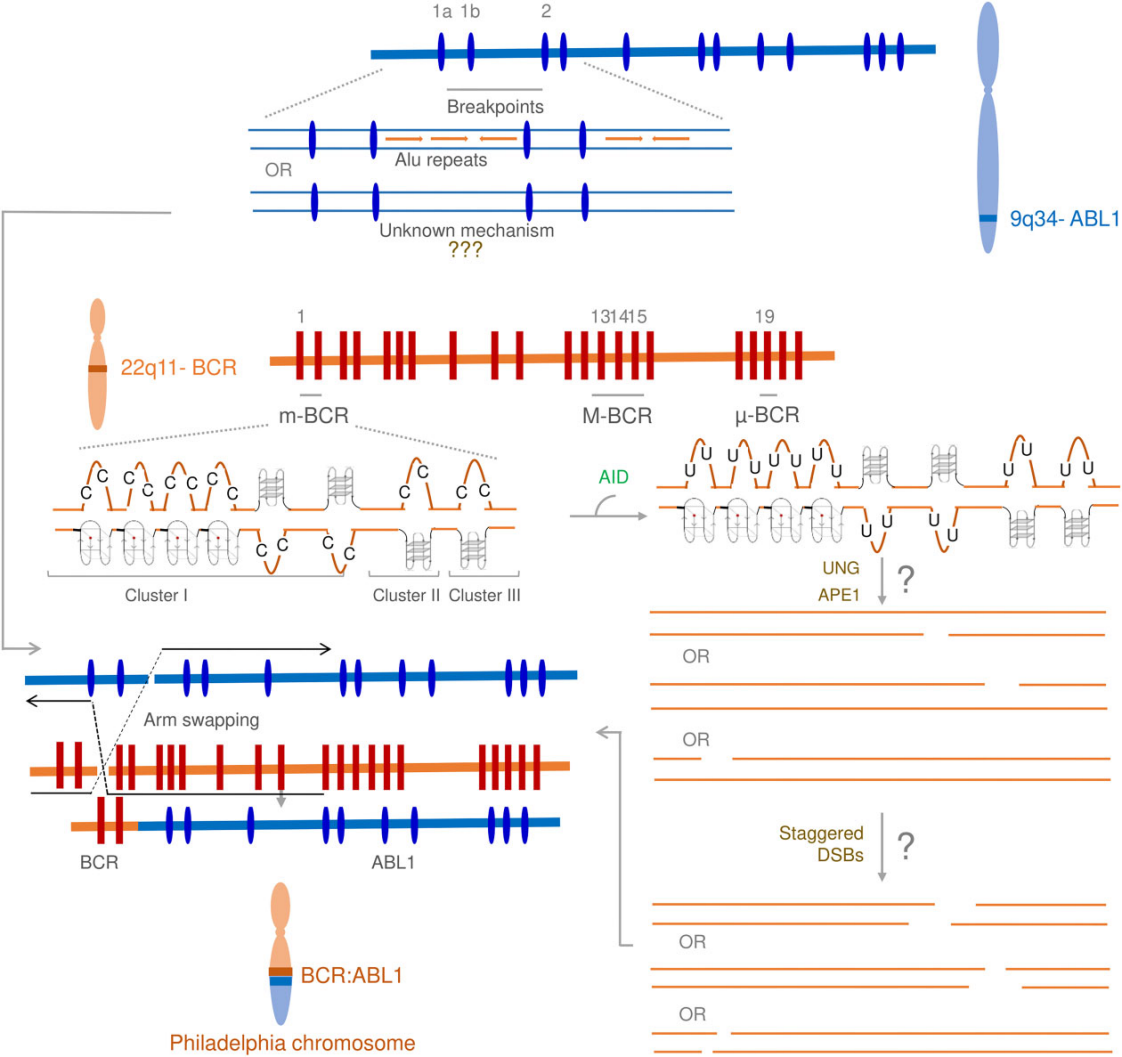
Model depicting the mechanism of action of AID leading to *BCR* fragility. Structure of the *BCR* and *ABL* genes with the breakpoint regions. The *ABL* gene contains one large breakpoint region, which is known to be facilitated due to the presence of Alu repeats, while other unknown mechanisms might be possible. Three breakpoint regions have been found in the *BCR* gene: m-bcr, M-bcr, and μ-bcr, which are associated with the p190, p210, and p230 BCR–ABL fusion proteins. The present study showed the formation of multiple G-quadruplexes in the *BCR* gene. AID can act on the single-stranded regions of these structures, converting cytosine to uracil and repairing the base excision. We speculate that UNG generates an abasic site, cleaved by APE1, generating single-stranded breaks. Aberrant repair of these breaks can lead to mutations. The DSBs generated on *BCR* on chromosome 9 and *ABL* on chromosome 22 may finally lead to t(9;22) translocation when there is arm swapping between chromosomes 9 and 22. The arm swapping between the chromosomes leads to the Philadelphia chromosome in CML patients.

Our study can explain the molecular mechanism of the fragility of *BCR* gene during the generation of the Philadelphia chromosome (Fig. 7). Our results provide evidence for the formation of multiple G-quadruplex structures at *BCR*. During physiological processes like replication or transcription, DNA unwinds and facilitates the formation of G-quadruplexes at *BCR*. The formation of these structures results in unpaired DNA, which can become direct targets for AID.

In the case of t(9;22) translocation, AID could be one of the major players contributing to fragility. AID can act on these single-stranded regions due to the formation of G-quadruplex structures and deaminate the unpaired cytosines to uracil. This is repaired by the base excision repair (BER) pathway where uracil is recognized by Uracil N Glycosylase (UNG), generating an abasic site, followed by an AP endonuclease (APE) cleavage. It may be possible that when there is a failure of BER, nicks can be converted into staggered DSBs during replication or due to a second break in the opposite strand at a proximity or during transcription. Finally, swapping of chromosomal arms result in *BCR/ABL* translocation, known as Philadelphia chromosome (Fig. 7).

Overall, our study delineates one potential mechanism of fragility in the *BCR* region. However, the formation of G-quadruplex at Cluster III has yet to be investigated in greater detail. Although the presence of Alu repeats is known to be one of the major reasons behind the breakage of the *ABL* region, more studies are required to understand the mechanisms behind the breakage of *ABL*.

## Supplementary Material

gkaf167_Supplemental_Files

## Data Availability

The data underlying this article are available in the article and its online supplementary material.
