# Supplementary material for: Formation of multiple G-quadruplexes contributes toward BCR fragility associated with chronic myelogenous leukemia
Source: Nucleic Acids Res. 2025 Mar 20;53(6):gkaf167. doi: 10.1093/nar/gkaf167 (PMC11925732; doi:10.1093/nar/gkaf167)

Supplementary Materials

Supplementary Figure Legends

Figure S1. Analysis of *BCR* region for G-quadruplex and patient breakpoints. Analysis and marking of the reported patient breakpoints in the *BCR* region divided into Cluster I, II, and III. The boxed region represents the predicted G quadruplex region. The blue box indicates the G4 motif in the (+) strand (GM3–GM7), while the orange box indicates the G4 motif in the (-) strand (GM1–GM2). The black arrow represents the breakpoint regions.

Figure S2. CD studies are to investigate G-quadruplex structure formation at the *BCR* GM2 region. A. CD spectra for the G-strand of the GM2 region in TE with and without KCl and was incubated at 37°C for 1 h. C-strand and mutant G-strands (M1, M2, and M3) were the controls. **B.** CD spectra for the G-strand of the GM2 region in TE in the presence of KCl at different temperatures (25, 37, 50, 60, 80, and 90ºC). **C.** CD spectra for the G-strand of the GM2 region in TE with KCl after incubating at 37°C for 1 h along with heat-denatured oligomer and after reforming the structure.

Figure S3. Polymerase arrest assay to evaluate G-quadruplex-forming region at the *BCR* breakpoint region. A. Schematic representation of the positions of primers and G-quadruplex motifs in the primer extension assay on plasmid DNA. GM1 to GM6 indicate the identified G-quadruplex motifs. Primers ET33, ET34, ET35, and ET36 were used for the primer extension study. **B.** Oligomers spanning regions GM1 and GM3 were annealed with respective radiolabeled primers in the presence of 50 mM KCl and were used for polymerase arrest assay. The extension reaction was performed using *Taq* DNA polymerase. **C.** The gel profile following primer extension on wild-type, mutant I (two stretches, 6 Gs were mutated) for both GM2 and GM3 regions of *BCR* region. Full-length extension products are marked, and pause sites are indicated as truncated products. A random oligomer with a GC content and sequence like the *HIF1*α promoter was used as negative and positive controls, respectively. Primer MN96 was used for *BCR* and random sequence, whereas MN119 was used for *HIF1*α region extension.

Figure S4. AID/BG4 ChIP of *BCR* and *in vitro* binding of AID to *BCR* fragile region. A. Sequencing of amplified fragments using *BCR* primers to confirm the region of interest from BG4 pulldown samples. **B.** Sequencing of amplified fragments to confirm the region of interest from AID pull-down samples. **C.** EMSA studies using purified AID to the *BCR* G-quadruplex forming region and a control region with no G4 DNA formation. The oligomers were heat denatured (to disrupt if any potential structures have formed) at boiling temperature and allowed for slow cooling in the presence of 100 mM KCl. Once the samples reached room temperature,

increasing concentrations (0, 20, 40, 60 nM) of purified AID were added and incubated for 1 h. The reaction was then loaded on a 6% native PAGE and ran at 100 V for 45 min. This figure represents the higher exposure of Figure 5D, emphasizing AID binding interactions with the BCR G-quadruplex structure (A lower exposure of the same gel is shown in the main text as Figure 5D).

Figure S5. Analysis of G-quadruplex structure formation at *BCR* cluster II (GM7). A. Sequence corresponding to GM7 of *BCR* cluster II with the positions of guanines (blue) and their mutation (red) indicated. **B.** Gel profile showing G4 DNA formation at GM7. G- and C-strand were loaded on native PAGE (15%) after incubation in the presence and absence of KCl in the reaction and running buffer.

Figure S6. DMS protection assay for the G-quadruplex-forming region at the *BCR* breakpoint region, GM2. A. Sequence corresponding to G2 region of *BCR* with the positions of guanines indicated. **B.** DMS protection assay for GM2 of *BCR* breakpoint region. The wild-type G-rich strand in TE in the presence and absence of 100 mM KCl at 37°C for 1 h were treated with DMS, followed by cleavage with piperidine, and were resolved on a 15% denaturing PAGE gel. **C.** Representative two-dimensional model of the intramolecular G-quadruplex structure formed at the *BCR* GM2 region. The position of guanines and other nucleotides involved in the structure are marked. Arrows indicate the orientation of the strands.

Figure S1

CCTCACTCTGCCGCCAGACCAGCACTGCACTTGAGAGCCAAGTGCCCTCTCCTGAGCCCCCAAGCCCTGG

CACCAGTTCTTGCCGTGCCCCTTCCCCAGGGTGTGTGGTGGAGGTCCAGTGGGAGGGGCCAGAGGGCCAA

GGAGCAGATTGACCAATTGGTGCACCTCTTTTCCAACCTCCCAGGTTTCAGAAGCTTCTCCCTGACATCC

Cluster I

GTGGAGCTGCAGATGCTGACCAACTCGTGT**G**TGAAACTCCAGACTGTCCACAGCATTCCGCTGACCATCA

GM3

ATAAGGAAGGTGGGCCCCCCCGTTTCCGTGTACAGGGCACCTGCAG**G**GAGGGCAGGCAGCTAGCCTGAAG

GM4

GCTGATCCCCCCTTCCTGTTAG**C**A**C**TTTTGATGGGACTAGTGGACTT**T**GGTTCAGAAGGAAGAGCTATGC

TTGTTAGGGCCTCTTGTCTCC**T**CCCAGGAGTGGACAAG**G**TGGG**T**TAGGAGCAGTTTCTCCCTGAGTGGCT

GM5

GM6

G**C**TGCTGGGTGGTTGAGGAG**A**T**G**CACGGCTTCTGTTCCTAGTCACAA**G**GCTGC**A**GC**A**GACGCTCCTCAGA

GM1

TGCTCTG**T**GCCTTGGATCTGGCCCCACTCCCGTCCTCCCAGCCCTCCTCTCCTCCAGCTACCT**G**CCAGCC

G**G**CACTTTTGGTCAAGCTGTTTTGCATTCACTGTTGCACATATGCTCAGT**C**ACACACACAGCATACGCTA

GM2

TGCACATGTGTCCACACACACCCCACCCACATCCCACATCACCCCGACCCCCTCTGCT**G**TCCTTGGAACC

TTATTACACTTCGAGTCACTGGTTTGCCTGTATTGTGAAACCAGC**TG**GATCCTGAGATCCCCA**AGA**CAGA

AATCAT**G**ATGAGTATGTTTTTGGCCCA**T**GACACTGGC**T**TACCT**T**G**T**GCCAGGCAGA**T**GGCAGCCACACAG

TGTCCACCGGATGG**T**TGATTTTGAAGCAGAGTT**A**GCTTGTCACCTGCCTCCCTTTCCCGGGACA**A**CAGAA

GCTGACCTCTTTGATCTCTTGCGCAGATGATGAGTCTCCGGGGCTCTATGGGTTTCTGAATGTCATCGTC

Cluster II

CACTCAGCCAC**T**GGATTTAAGCAGAGTTCAA**GT**AAGTACTGGTTTGGGGAGGAGGGTTGCAGCGGCCGAG

GM7

CCAGGGTCTCCACCCAGGAAGGACTCATCGGGCAGGGT**GT**GGGGAAACAGGGAGGTTGTTCAGATGACCA

CGGGACACCTTTGACCCTGGCC**G**CTGTGGAGTGTT**T**G**T**GCTGGTTGATGCCTTCTGGGTGTG**G**AATTGTT

TTTCCCGGAGTGGCCTCTGCCCTCTCCCCTAGCCTGTCTCAG**A**TC**C**TGGGAGCTGGTGAGCTGCCC**C**CTG

CAGGTGGATCGAGTAATTGCAGGGGTTTGGCAAG**G**ACTTTGACAGACATCCCCAGGGGT**G**CCCGGGAGTG

TGGGGTCCAAGCCAGGAGGGCTGTCAGCAGTGCACCTTCACCCCACAGCAGAGCAGATTTGGCTGCTCTG

Cluster III

TCGAGCTG**G**ATGGATAC**T**ACTTTTTTTTTCCTTTCCCTCTAAGTGGGGGTCTCCCCCAGCTACTGGAGCT

GTCAGAACAG**T**GAAGGCTGGTAACACATGAGTTGCACTGTGTAAGTTTCTC**G**AGG**C**CGGGCGCAGTGGCT

ATGCCTGTAATCCCAGCACTTTGGGA**G**GCTGAGGCAGGTGGATCGCTTGAGCTCAGGAGTTGGAGACCA

GCCTGACCAACATGGTGAAACCCTGTGTCTACTAAAAATACAAAGATTAGCCGGGCTAG**G**CAGT**G**GGCAC

CTGTAATCACAACTGCTTGGGAGGCTGAGGGAAGAGAATCGCTTGAA**C**CCAG**G**AGGCGGAGGTTGC**A**GTG

AGCCGAGCTTGTGCCACTGCATTCCAGCCTGGGCGACA**G**AGCAAGACTCCGCCTCAAAAAAAAAAAAAA

AAGTTCCTAGAAACAGCAAAATGTGGAGACAGAAAGCTTACCAGGGATTGTTGGGGAATGGGGTTGGGAG

Figure S2

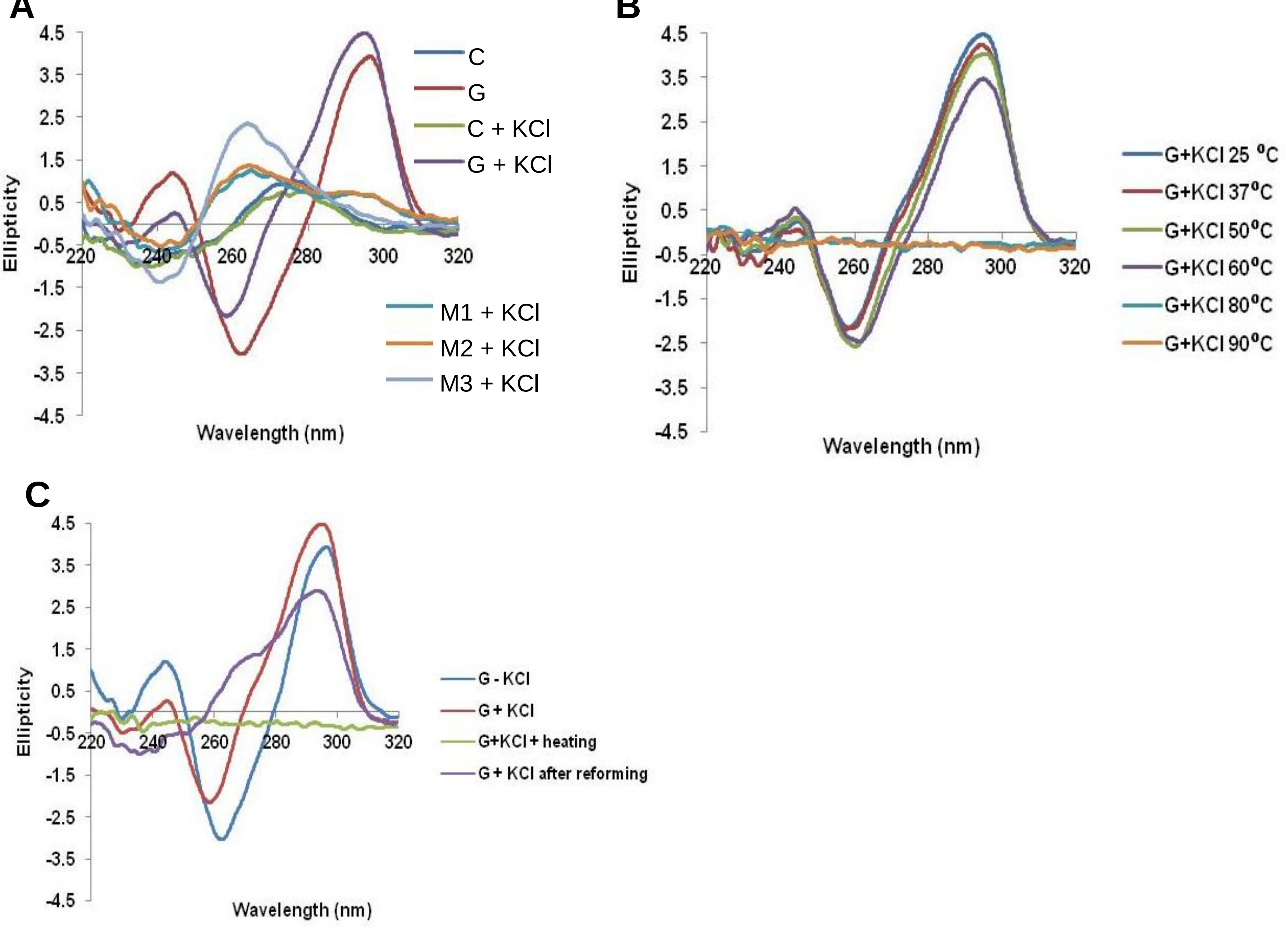
A
Ellipticity
Wavelength (nm)
4.5
3.5
2.5
1.5
0.5
-0.5
-1.5
-2.5
-3.5
-4.5
220
240
260
280
300
320
C
G
C + KCl
G + KCl
M1 + KCl
M2 + KCl
M3 + KCl
B
G+KCl 25 °C
G+KCl 37°C
G+KCl 50°C
G+KCl 60°C
G+KCl 80°C
G+KCl 90°C
C
G - KCl
G + KCl
G+KCl + heating
G + KCl after reforming

Figure S3

A

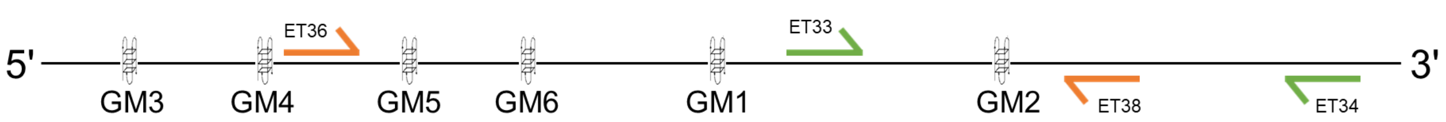

B

WT1 5′– GAGGGGGTCGGGGTGATGTGGGATGTGGGTGGGGTGTGTCAACTCTCTAAAGGCAGCGTC –3′
Mut1 5′– GAGGGGGTCAATGTGATGTTCAATGTGGGTGGGGTGTGTCAACTCTCTAAAGGCAGCGTC –3′
WT2 5′– ATCGGGCAGGGTGTGGGGAAACAGGGAGGTTGTTCACAACTCTCTAAAGGCAGCGTC –3′
Mut2 5′– ATCGGGCAACATGTAATGAAACAGGGAGGTTGTTCACAACTCTCTAAAGGCAGCGTC –3′
RN 5′– GCGGAGCGAGCCGAGCGAGCGTGTGAGGTTGAGTGCGTTGAAACAGGCCACGTAAAGCAACTCTCTAAAGGCAGCGTC –3′
PC 5′– TCCAACTATGTATACGCGCGGGGAGGGGAGAGGGGCGGGAGCGCGTTAGCGACACGCAATTGCTATAGTGAGTCGTATTA –3′

C

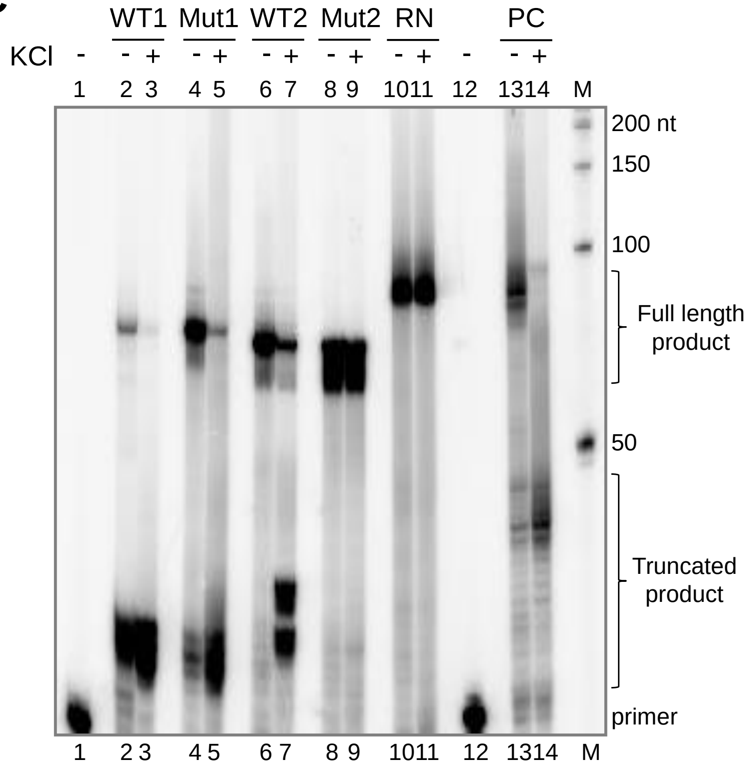

Figure S4

A

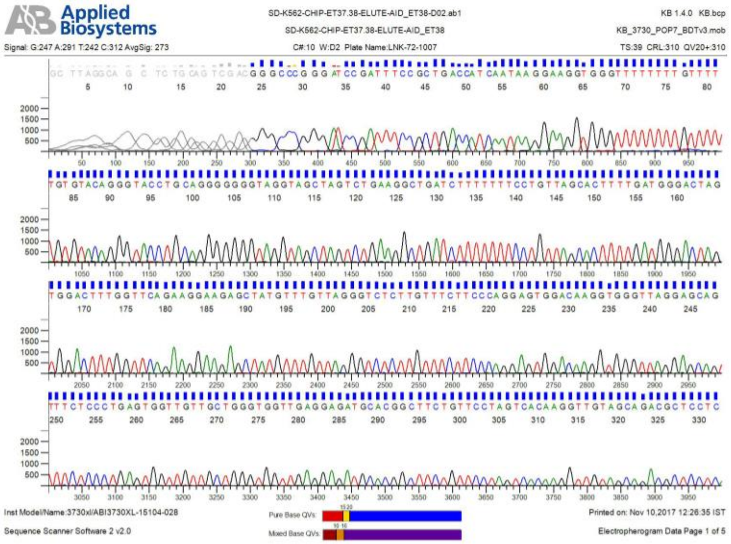

B

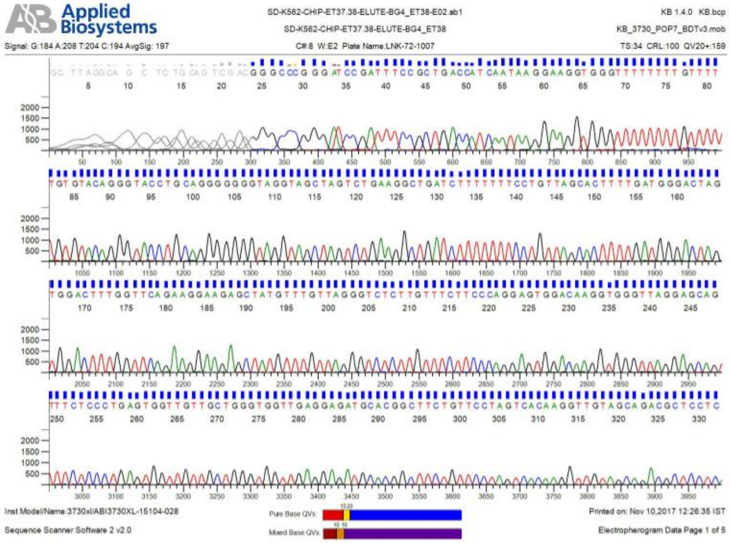

C

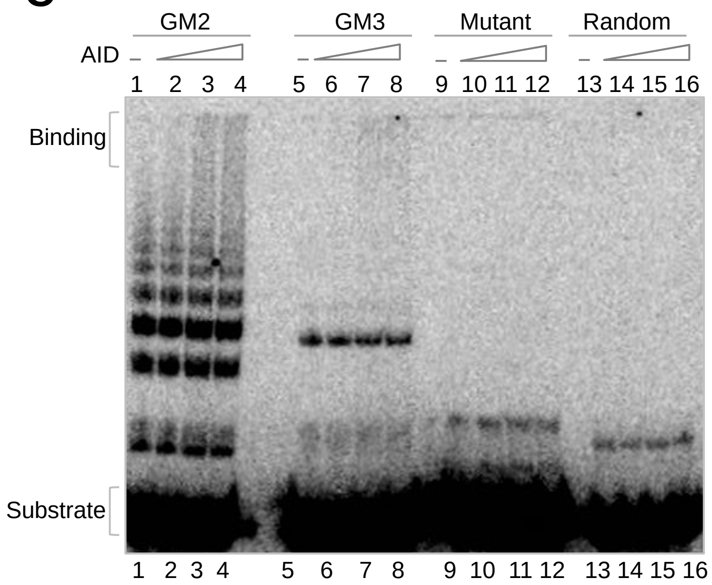

Figure S5

A

C 5′-CCCTGTTTCCCCACACCCTGCCC-3′

G 5′-GGGCAGGGTGTGGGGAAACAGGG-3′

B

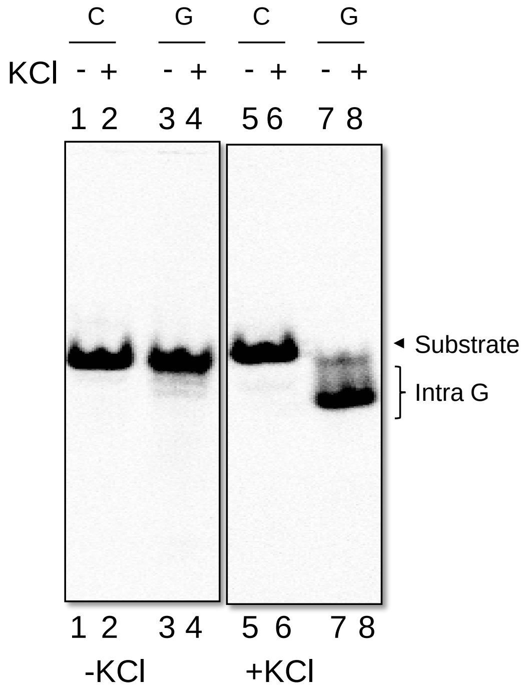

Figure S6

A

GM2: 5′-G_1CAG_2A$G_3G_4G_5G_6G_7$TC$G_8G_9G_{10}G_{11}$TG_{12}ATG_{13}T$G_{14}G_{15}G_{16}$ATG_{17}T$G_{18}G_{19}G_{20}$TG_{21}-3′

B

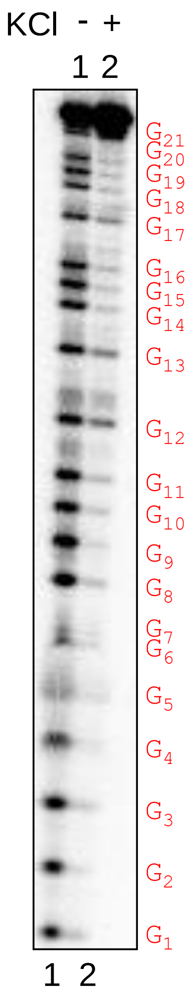

C

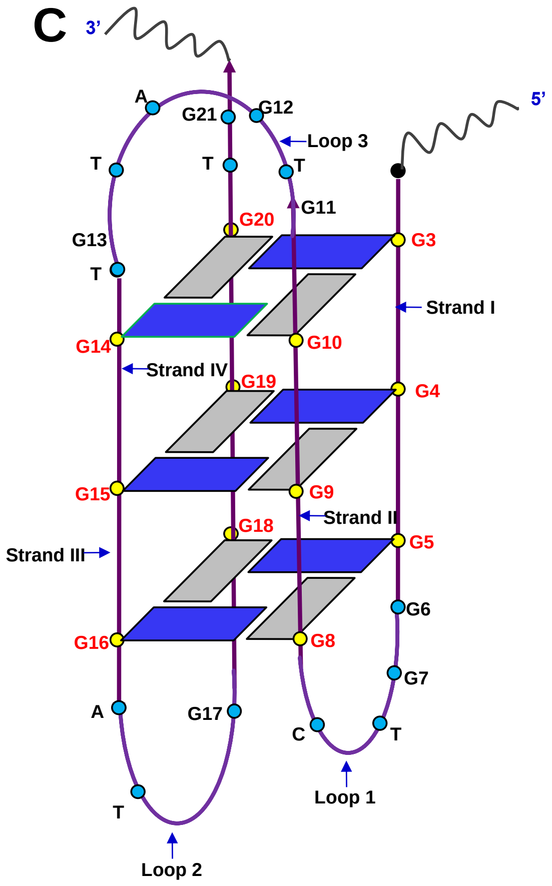

Supplement: gkaf167_Supplemental_Files [file gkaf167_supplemental_files.zip › SCR_BCRABL_SupplText_Figures.pdf]
