# Supplementary material for: Formation of multiple G-quadruplexes contributes toward BCR fragility associated with chronic myelogenous leukemia
Source: Nucleic Acids Res. 2025 Mar 20;53(6):gkaf167. doi: 10.1093/nar/gkaf167 (PMC11925732; doi:10.1093/nar/gkaf167)
Supplement: gkaf167_Supplemental_Files [file gkaf167_supplemental_files.zip › Table S1v05R2.doc]

| S.No | Oligomer | Sequence |
| --- | --- | --- |
| 1 | WT1/ET19 | 5’-GAGGGGGTCGGGGTGATGTGGGATGTGGGTGGGGTGTGTCAACTCTCTAAAG-GCAGCGTC-3’ |
| 2 | Mut1/ET20 | 5’-GAGGGGGTCAATGTGATGTTCAATGTGGGTGGGGTGTGTCAACTCTCTAAAG-GCAGCGTC-3’ |
| 3 | WT2/ET21 | 5’-ATCGGGCAGGGTGTGGGGAAACAGGGAGGTTGTTCACAACTCTCTAAAGGCA-GCGTC-3’ |
| 4 | Mut2/ET22 | 5’-ATCGGGCAACATGTAATGAAACAGGGAGGTTGTTCACAACTCTCTAAAGG-CAGCGTC-3’ |
| 5 | RN | 5’-GCGGAGCGAGCCGAGCGAGCGTGTGAGGTTGAGTGCGTTGAAACAGGCCAC-GTAAAGCAACTCTCTAAAGGCAGCGTC-3’ |
| 6 | PC | 5’-TCCAACTATGTATACGCGCGGGGAGGGGAGAGGGGCGGGAGCGCGTTAGCG-ACACGCAATTGCTATAGTGAGTCGTATTA-3’ |
| 7 | GM1C | 5’-CCCCACTCCCGTCCTCCCAGCCC-3’ |
| 8 | GM1G | 5’-GGGCTGGGAGGACGGGAGTGGGG-3’ |
| 9 | GM2C | 5’-CCCACATCCCACATCACCCCGACCCCC-3’ |
| 10 | GM2G | 5’-GGGGGTCGGGGTGATGTGGGATGTGGG-3 |
| 11 | GM3C | 5’-CCTGCCCTCCCTGCAGGTGCCC-3’ |
| 12 | GM3G | 5’-GGGCACCTGCAGGGAGGGCAGG-3’ |
| 13 | GM4C | 5'-CCTTCTGAACCAAAGTCCACTAGTCCC-3’ |
| 14 | GM4G | 5'-GGGACTAGTGGACTTTGGTTCAGAAGG-3' |
| 15 | GM5C | 5'-CCTAACCCACCTTGTCC-3' |
| 16 | GM5G | 5'-GGACAAGGTGGGTTAGG-3' |
| 17 | GM6C | 5'-CCGTGCATCTCCTCAACCACC-3' |
| 18 | GM6G | 5’-GGGTGGTTGAGGAGATGCACGG -3’ |
| 19 | GM7C | 5’-CCCTGTTTCCCCACACCCTGCCC-3’ |
| 20 | GM7G | 5’–GGGCAGGGTGTGGGGAAACAGGG–3’ |
| 21 | 2M1 | 5’-GGGGGTCGGGGTGATGTGGGATGTATA-3’ |
| 22 | 2M2 | 5’-GGGGGTCGGGGTGATGTTCAATGTGGG-3’ |
| 23 | 2M3 | 5’-GGGGGTCAATGTGATGTGGGATGTGGG-3’ |
| 24 | 7M1 | 5’-GGGCAGGGTGTGGGGAAACAATC-3’ |
| 25 | 7M2 | 5’-GGGCAGGGTGTAATGAAACAGGG-3’ |
| 26 | 7M3 | 5’-GGGCAACATGTGGGGAAACAGGG-3’ |
| 27 | SB1 | 5’-GATCCCTCTACCGGTACTACTCGAGCCGGCTAGCTTCGATGCAGCCTGAG-3’ |

**Table S1.** List of oligomers used in the study
